# Landscape Features Shape Maternal Genetic Structure of Asian Elephants in Thailand: Insights from mtDNA

**DOI:** 10.3390/biology15040358

**Published:** 2026-02-20

**Authors:** Supansa Rerkdee, Worapong Singchat, Thitipong Panthum, Trifan Budi, Warong Suksavate, Pannita Neepai, Aingorn Chaiyes, Thiti Sornsa, Wichanon Saenphala, Boripat Siriaroonrat, Kornsorn Srikulnath, Prateep Duengkae

**Affiliations:** 1 Special Research Unit for Wildlife Genomics (SRUWG), Department of Forest Biology, Faculty of Forestry, Kasetsart University, Bangkok 10900, Thailand; supansa.re@ku.th (S.R.); worapong.singc@ku.ac.th (W.S.); thitipong.pant@ku.ac.th (T.P.); fforwos@ku.ac.th (W.S.); pannita.nee@ku.th (P.N.); 2 Animal Genomics and Bioresource Research Unit (AGB Research Unit), Faculty of Science, Kasetsart University, Bangkok 10900, Thailand; trifan.bu@ku.th (T.B.); aingorn.ch@ku.ac.th (A.C.); 3 The International Undergraduate Program in Bioscience and Technology, Faculty of Science, Kasetsart University, Bangkok 10900, Thailand; 4 Chachoengsao Wildlife Research Station, Department of National Parks, Wildlife and Plant Conservation, Chachoengsao 24160, Thailand; thiti.sornsa@gmail.com; 5 Phu Khieo Wildlife Sanctuary, Department of National Parks, Wildlife and Plant Conservation, Khon San 36110, Thailand; pattanurug@gmail.com; 6 Faculty of Environment and Resource Studies, Mahidol University, Nakhon Pathom 73170, Thailand; boripat.siriaroonrat@gmail.com; 7 Biodiversity Center Kasetsart University (BDCKU), Bangkok 10900, Thailand

**Keywords:** Asian elephant, D-loop, landscape genetics, resistance surface, habitat fragmentation

## Abstract

Landscape features often shape maternal genetic structure by influencing connectivity. In this study, habitat fragmentation, a major threat to the Asian elephant (*Elephas maximus*), was assessed through an integrated approach involving mitochondrial DNA (mtDNA), habitat-suitability modeling, and circuit-based landscape-resistance analyses. Two regions from Thailand, Phu Khieo (PK) and Khao Ang Rue Nai (ARN) Wildlife Sanctuaries, were investigated. Fourteen mtDNA haplotypes were identified among 66 samples, with relatively high diversity and population expansion in PK. Maternal genetic differentiation was identified between the PK and ARN groups. Environmental variables, such as urbanization and road proximity in ARN and topographic wetness and stream distance in PK, were associated with genetic distances, suggesting these features restricted female-mediated connectivity. Fine-scale spatial analysis revealed significant local genetic structure only in PK, whereas no autocorrelation was detected in ARN, suggesting potential impacts of fragmentation. Genetic landscape surfaces illustrated spatial heterogeneity, pinpointing isolation zones near high anthropogenic disturbance. These findings demonstrate that broad-scale models may overlook fine-scale patterns of maternal genetic isolation. Therefore, conservation strategies should incorporate spatially explicit analyses to identify and restore crucial movement corridors, particularly in fragmented regions like ARN, for promoting connectivity and population viability.

## Simple Summary

Forest fragmentation is making it harder for elephants to move and survive in the long term. This study aimed to understand how landscape features are associated with elephant movement in two wildlife sanctuaries in Thailand, Phu Khieo and Khao Ang Rue Nai. We combined information from elephant feces with mapping tools to study movement patterns. The results show that elephants in these areas exhibit genetic differences that align with restricted movement routes. In Khao Ang Rue Nai, roads and expanding human areas are strongly linked to these barriers, while in Phu Khieo, steep hills and rivers appear to shape elephant movement. This study provides a practical tool to identify areas where elephants are isolated. The findings can help guide habitat restoration, improve forest connections, and reduce future conflicts between elephants and nearby communities.

## 1. Introduction

Habitat fragmentation is a primary driver of global biodiversity loss, threatening the long-term survival of wide-ranging species through the disruption of metapopulation dynamics [1,2]. Isolated subpopulations become vulnerable to demographic stochasticity and genetic drift, potentially leading to inbreeding depression and local extinction if connectivity, and thus gene flow, is lost [3,4]. The Asian elephant (*Elephas maximus*), the largest terrestrial mammal in Asia, is a keystone species, which maintains tropical forest ecosystems by dispersing seeds, modifying vegetation, and creating microhabitats, such as water holes [5]. Their survival has, however, been threatened by habitat loss, fragmentation, and conflicts with humans, resulting in >50% reduction in populations since the mid-20th century [6]. In Thailand, elephant habitats, which were once continuous, have contracted into isolated patches, confining an estimated 4000 individuals within the fragmented protected areas [7]. Habitat fragmentation associated with agricultural expansion, infrastructure development, and urbanization has likely altered landscape connectivity, potentially influencing dispersal patterns and population structure. This has resulted in limited access to resources, mates, which reduces effective dispersal and gene flow. Over time, this contributes to genetic isolation through demographic effects such as a reduced effective population size (*N*_e_) and increased genetic drift [8,9,10]. As a consequence, small populations, which are prone to inbreeding depression and local extinctions, suffer from impaired gene flow, reduced genetic diversity, and an increased risk of extinction [8,11,12]. By the 1970s, elephants were largely extirpated from Thailand’s central plains owing to land conversion for agriculture and canals, leaving populations in mountainous regions [13]. Human settlements and expanding road networks also create landscape resistance by fragmenting habitats and constraining elephant movement [14]. High-resistance landscapes, which act as barriers, are often associated with increased genetic divergence and reduced recolonization opportunities. Habitat connectivity has been linked to elephant distribution [15]; however, the specific relationship between landscape resistance and genetic structure in Thailand remains largely unclear. Exploring these associations is crucial for developing effective conservation strategies for elephants in Thailand.

The concept of landscape resistance provides a valuable framework for understanding how environmental and anthropogenic activities influence animal movements [16]. Areas with high resistance, such as steep or heavily modified terrain, are obstacles to dispersal, whereas relatively highly permeable habitats facilitate movement [14]. Circuit theory is increasingly used to model landscape connectivity by simulating animal movement as an electrical current flowing through resistance surfaces [17,18]. These models often identify narrow routes, in which movement is concentrated owing to limited surrounding permeability. Such features offer insights into the spatial constraints on movement and help prioritize conservation efforts in fragmented habitats [19]. Therefore, the understanding of these landscape features is essential for developing effective strategies to maintain connectivity and support species survival. In addition to spatial modeling, mitochondrial DNA (mtDNA) analyses provide complementary insights into how landscape features influence population structure. Maternal inheritance and high mutation rate of mtDNA enable tracing of lineage-specific dispersal, historical bottlenecks, and long-term connectivity [20,21]. It is important to recognize that mtDNA offers valuable insights into genetic data, but it specifically reflects maternal lineages, providing a more focused perspective [22]. In Asian elephants, different haplogroups across Southeast Asia reflect ancient migration routes and biogeographic barriers [23]. Significant divergence of mtDNA between populations separated by highways and agricultural areas has been reported in India and Sri Lanka [24]. However, such genetic studies on Asian elephants are scarce in Thailand [11,25], where habitat changes may have shaped genetic diversity and threaten population resilience.

Two key elephant habitats in Thailand, Phu Khieo (PK) and Khao Ang Rue Nai (ARN) Wildlife Sanctuaries, are characterized by contrasting landscapes and levels of human–elephant conflict. PK in northeastern Thailand features a largely continuous upland forest with minimal disturbance, supporting natural movement, and estimates a population of 114 wild elephants based on camera-trap surveys conducted between 2016–2019 [26]. By contrast, ARN in the east, has experienced extensive forest loss owing to agriculture and roads, resulting in highly fragmented landscapes intersected by farmland and infrastructure, and supporting an estimated 79–334 elephants based on camera-trap surveys conducted between March 2017 and 2018 [27]. These landscape differences influence elephant movement and maternal lineage patterns, with PK supporting movement in continuous habitats and ARN supporting movement in putatively fragmented landscapes. Instances of human-elephant conflict have been reported in both PK and ARN, with relatively highly frequent and severe incidents in ARN owing to its proximity to agricultural zones [28]. These conditions have disrupted historical migration routes, leading elephants to suboptimal habitats, and potentially increasing their spatial isolation. Historically, PK and ARN were parts of a broader and contiguous forest landscape until the mid-20th century; however, connectivity has become restricted to narrow or degraded pathways owing to land-use change and human development [29]. Relatively high landscape resistance is hypothesized to be present in ARN owing to extensive habitat fragmentation and anthropogenic development, which may shape maternal genetic structure and limit sharing of mtDNA haplotypes with PK. Landscape features are considered to function as primary genetic barriers, structuring genetic variation both between and within populations.

This study aimed to fill this knowledge gap by explicitly assessing the relationship between landscape resistance and maternal genetic structure (mtDNA haplotype differentiation) in two ecologically contrasting elephant populations in Thailand. Our primary objectives were to: (1) characterize maternal haplotype diversity and distribution within and between the two sanctuaries; (2) develop a landscape resistance model based on environmental and anthropogenic variables; and (3) assess the relationship between genetic divergence and both geographic distance (isolation by distance, IBD) and landscape resistance (isolation by resistance, IBR) to identify the specific landscape features associated with genetic isolation. We hypothesize that anthropogenic factors, such as major roads and settlements, represent the primary resistance features correlated with maternal lineage differentiation between PK and ARN.

## 2. Materials and Methods

### 2.1. Study Areas

This study was conducted in two protected areas in Thailand: Phu Khieo Wildlife Sanctuary (PK) in Chaiyaphum Province (16°05′35″ N, 101°20′55″ E) and Khao Ang Rue Nai Wildlife Sanctuary (ARN) in Chachoengsao Province (13°12′00″ N, 101°44′00″ E) (Figure S1). These areas with contrasting landscape contexts were chosen to evaluate the influence of environmental resistance on maternal gene flow in wild Asian elephants. PK, located in northeastern Thailand, is characterized by a largely intact forest ecosystem with minimal anthropogenic disturbance, featuring interconnected ridgetop plains and valleys that function as natural corridors for facilitating elephant movement, despite locally restricted foraging on steep slopes. In contrast, ARN in eastern Thailand has experienced notable habitat decline to approximately 30% owing to agriculture and infrastructure development, resulting in fragmented patches intersected by roads, such as Highway 3076. Field surveys for this study covered the entire interior and periphery of both sanctuaries. However, the ecological context of ARN is distinct; it is recognized as the primary zone for Human-Elephant Conflict in Thailand, where elephants exhibit strong behavioral tendencies to expand their range outside protected boundaries [30]. This landscape is heavily influenced by seasonal agricultural activities, particularly during the sugarcane harvest, where road networks serve as high-activity zones due to the presence of crop transport trucks. The differences in habitat continuity, elevation, and human impact between PK and ARN may provide a strong comparative framework for understanding how landscape features influence gene flow in elephant populations.

### 2.2. Specimen Collection

Fecal samples were noninvasively collected from wild elephants from PK and ARN. Permission for sample collection was granted under DNP Permit Numbers 6501.0901/0519 for PK and 0907.404/27069 for ARN. Sampling was conducted during the dry season from November 2023 to May 2024, a period when elephant movement is relatively predictable owing to scarcity of water sources [28]. Fecal samples were collected along the daily movement routes typically used by elephants, particularly in the early morning when fresh dung piles are relatively likely to be found and identified. To increase the likelihood of sampling distinct individuals, collection efforts were conducted along established patrol routes used by park officials. Although this approach restricted access to more remote or off-trail areas, it effectively focused sampling on the main movement corridors used by the elephant population. This strategy helped maximize our chances of sampling different individuals, as these routes are frequently used by various elephant groups. To assess the freshness of samples, each fecal pile was examined for texture, moisture, and odor, which allowed for the inclusion of both fresh and moderately aged samples. Freshly deposited fecal piles were prioritized to obtain the surface mucosal layer that is rich in host epithelial cells [31]. Fecal piles were spaced at least 1-m apart for minimizing the chance of sampling the same individual multiple times. Fecal samples were collected from piles spaced at least 1 m apart to minimize repeated sampling of the same individual; however, this distance does not definitively guarantee that samples originated from unique individuals. Each sample was placed in a sterile 50-mL polypropylene centrifuge tube (Corning™, Corning Inc., New York, NY, USA) containing 95% ethanol, labeled with a unique identity and global positioning system coordinates, and was stored at 4 °C before being transported to the laboratory for storage at −20 °C until DNA extraction. All experimental procedures were approved by the Kasetsart University Animal Experiment Committee (approval number: ACKU63-SCI-017) and were conducted in accordance with the Regulations on Animal Experiments at Kasetsart University and ARRIVE Guidelines (https://arriveguidelines.org).

### 2.3. DNA Extraction, Amplification, and Sequence Processing

Genomic DNA was extracted using a ZymoBIOMICS DNA Miniprep Kit (Zymo Research, Irvine, CA, USA) with slight modifications, including 1-h lysis at 55 °C and 40 min of continuous bead beating using a Vortex Genie (Scientific Industries, Bohemia, NY, USA) with 2-mL BashingBead tubes. DNA concentration and purity were measured using a NanoDrop 2000 spectrophotometer (Thermo Fisher Scientific, Waltham, MA, USA), and DNA quality was verified using 1% agarose gel electrophoresis. The partial mitochondrial D-loop region was amplified using the primers MDL5 (5′-TTACATGAATTGGCAGCCAACCAG-3′) and MDL3 (5′-CCCACAATTAATGGGCCCGGAGCG-3′) [20]. Each 15-μL polymerase chain reaction (PCR) mixture contained 1× ThermoPol buffer, 1.5 mM MgCl_2_, 0.2 mM dNTPs, 0.5 μM primers, and 0.5 U Taq polymerase (Apsalagen Co., Ltd., Bangkok, Thailand). PCR amplification was carried out using the following conditions: initial denaturation at 94 °C for 3 min; 40 cycles at 94 °C for 45 s, 63 °C for 45 s, 72 °C for 30 s; and a final extension at 72 °C for 10 min. The PCR products were visualized on 1% agarose gels and purified using a MEGAquick-spin Plus kit (iNtRON Biotechnology, Seongnam, Republic of Korea). Purified PCR products were subjected to bidirectional Sanger sequencing using an ABI 3730XL Automatic Sequencer (Applied Biosystems, Foster City, CA, USA) at Macrogen Inc. (Seoul, Republic of Korea). Bidirectional chromatograms were assembled and quality-checked using Geneious Prime version 2025.1 (https://www.geneious.com), aligned using ClustalW in MEGA version 11 [32], and trimmed to 359 bp. All unalignable and gap-containing sites were carefully removed and manually trimmed from the datasets. Haplotype screening was performed using DnaSP version 6.12.03 [33]. BLASTn (Available online: http://blast.ncbi.nlm.nih.gov/Blast.cgi, accessed on 14 June 2025) was used to search for nucleotide sequences in the National Center for Biotechnology Information database for confirming the identity of the amplified DNA fragments. A total of 459 mitochondrial DNA (mtDNA) D-loop sequences were analyzed, comprising 66 newly generated sequences from wild elephants in PK (n = 32) and ARN (n = 34), which have been deposited in GenBank under accession numbers PV649948–PV650013. The remaining 393 sequences were obtained from published sources and are listed in Table S1. Haplogroups α and β were assigned based on published reference sequences curated as previously described [13].

### 2.4. Genetic Diversity, Population Structure, and Haplotype Relationships

The mitochondrial genetic diversity and population structure indices were calculated as previously described [8]. Haplotype diversity (*h*), nucleotide diversity (*π*), and the average number of nucleotide differences were calculated using DnaSP version 6.12.03 [33] to quantify within and between population diversity. The average number of nucleotide substitutions per site between populations (*D*_xy_) and net nucleotide divergence (*D*_a_) were also estimated in DnaSP to evaluate inter-population genetic divergence. Nei’s genetic differentiation coefficient (*G*_ST_), Wright’s fixation index (*F*_ST_), molecular variance-based Φ_ST_, and gene flow (*N*_m_) from sequence and haplotype data were estimated using Arlequin version 3.5.2.2 [34]. *F*_ST_ and Φ_ST_ values were calculated based on 1000 permutations of haplotypes between populations, as previously described [35]. The statistical significance of these indices was assessed using permutation tests, in which *p*-values < 0.05 were regarded significant. Exact *p*-values are reported for transparency. Given the exploratory nature of the analyses and partially correlated tests, Bonferroni correction was not applied. *F*_ST_ measures differences in haplotype frequencies and Φ_ST_ incorporates evolutionary distances between haplotypes [36]. *F*_ST_ values were interpreted according to Wright’s (1978) thresholds [37], in which 0–0.05, 0.05–0.15, 0.15–0.25, and >0.25 indicate low, moderate, high, and very high genetic differentiation, respectively. The number of migrants per generation (*N*_m_) was estimated from *F*_ST_ values to infer effective maternal gene flow between populations. *N*_m_ values > 1 were considered indicative of sufficient gene flow. Analysis of Molecular Variance (AMOVA) was performed to partition genetic variations within and between populations. Relationships between mitochondrial haplotypes were visualized through a median-joining network constructed in PopART version 1.7 [38], with node sizes proportional to haplotype frequency, for inferring patterns of maternal lineage connectivity.

### 2.5. Phylogenetic and Demographic Inference

Phylogenetic relationships were inferred using IQ-TREE version 2.4.0 (Available online: http://iqtree.cibiv.univie.ac.at, accessed on 16 June 2025) [39], with all 459 sequences. ModelFinder integrated within IQ-TREE identified TIM3+F+G4 as the best-fit substitution model based on the Bayesian Information Criterion (BIC = 8379.102), ensuring optimal model selection for tree reconstruction [40]. A Maximum Likelihood (ML) tree was reconstructed with 1000 ultrafast bootstrap and 1000 SH-aLRT replicates for evaluating branch support. The resulting tree was manually rooted and annotated in Interactive Tree of Life version 7.2 (Available online: https://itol.embl.de/, accessed on 16 June 2025) [41]. Outgroup rooting used five taxa, including *Loxodonta africana* (African savanna elephant; AY742802), *Elephas antiquus* (straight-tusked elephant; KY499555), *Mammuthus primigenius* (woolly mammoth; MF770243), *Mammuthus columbi* (Columbian mammoth; KX027513), and *Mammuthus jeffersonii* (Jefferson’s mammoth; KX027559), for representing evolutionary divergence within Elephantidae and closely related lineages, thereby providing an appropriate reference for rooting and clustering Asian elephant haplotypes. To investigate the population demographic history and assess evidence of expansion, three complementary approaches based on mtDNA sequence data were applied. Neutrality tests, including Tajima’s *D*, Fu’s *Fs*, and Fu and Li’s D* and F*, were calculated in DnaSP version 6.12.03 [33] using population-specific alignments, as these statistics are sensitive to deviations from mutation–drift equilibrium that may indicate demographic events. Mismatch distribution and Harpending’s raggedness index were calculated using Arlequin version 3.5.2.2 [34], with the PK and ARN populations analyzed independently using the sudden expansion model. The interpretation criteria were followed as previously described [42]: a unimodal mismatch curve, low raggedness index, and nonsignificant sum of squared deviations (SSD) indicated recent population expansion, whereas multimodal curves with significant statistics suggested population stability or a complex demographic history. Bayesian Skyline Plot (BSP) was analyzed using BEAST version 1.10.4 [43] for inferring changes in effective population size (*N*_e_) over time. As the TIM3+F+G4 model identified by IQ-TREE was not available in BEAST, the closely related GTR+G model (BIC = 8383.538) was chosen as a suitable alternative. The model settings were configured in BEAUti [44]. A strict molecular clock was applied with a substitution rate of 0.406 × 10^−9^ substitutions/site/year for ensuring consistency with phylogenetic and paleogenomic studies [45]. Population-specific alignments for PK and ARN were independently analyzed, each in separate Markov chain Monte Carlo (MCMC) chains for 100 million generations, sampling every 1000 steps and discarding the first 10% as burn-in. The convergence of MCMC chains (effective sample size (ESS) > 200) was assessed, and the BSP was visualized in Tracer version 1.7.2 [46], following best practices in coalescent-based demographic inference.

### 2.6. Habitat Suitability Modeling and Construction of Resistance Surface

Habitat suitability models were developed using Maximum Entropy modeling in MaxEnt version 3.4.1 (Available online: https://biodiversityinformatics.amnh.org/open_source/maxent/, accessed on 20 June 2025) [47,48] for predicting the spatial distribution of suitable elephant habitats as a function of environmental variables and elephant presence records. The response variable was derived from field-verified fecal samples, making this a presence-only dataset. Twelve environmental variables were selected based on ecological rationale and previous studies on landscape genetics [49], comprising both natural features (canopy height, elevation, slope, forest type, terrain ruggedness index (TRI), topographic wetness index (TWI), percent tree cover, and distance to streams) and anthropogenic factors (distance to roads, nighttime lights, urbanization, and human population density) (Table S2). Raster data for all variables were standardized and resampled to a 30-m resolution using QGIS version 3.28, to ensure spatial comparability and alignment among layers [50]. Model performance was evaluated using the Area Under the Curve (AUC) metric, which ranges between 0–1, with high values indicating good performance. AUC values closer to 1 were considered to reflect high model accuracy. The inherent limitations of relying solely on AUC for presence-only data are acknowledged, as this metric can be inflated by the choice of background points and may not fully reflect true predictive discrimination [51]. To guard against overfitting, ensure a more comprehensive evaluation of model reliability, a leave-one-out cross-validation approach was implemented to assess model stability, and omission rates, indicating the proportion of presence localities predicted to be unsuitable, were calculated to evaluate predictive accuracy. The relative importance of individual variables in predicting elephant occurrence was determined using jackknife tests of regularized training gain. Each variable was separately modeled in MaxEnt for generating habitat suitability scores ranging from 0 (unsuitable) to 1 (highly suitable). Following widely adopted approaches in landscape studies [16], these scores were then reclassified and inverted using a linear transformation as follows:

R = 100 × (1 − S),(1)

where R is the resistance value assigned to each raster cell, and S is the original habitat suitability score. Univariate resistance surfaces were produced by this transformation, which were scaled from 1, indicating minimal resistance to movement, to 100, representing maximum resistance or a complete barrier, ensuring that areas identified as highly suitable for the species correspond to low resistance values, and vice versa. This standardization facilitates integration with circuit-based connectivity analyses (e.g., Circuitscape) and allows for comparability across studies [17]. This transformation method applied a uniform weighting across environmental gradients and did not account for the varying influence of each variable on species movement, assuming a linear and equal relationship between suitability and resistance. Habitat suitability was modeled independently for each site using occurrence points derived from field-verified fecal-sample presence data (PK, 133; ARN, 192) and site-specific environmental layers. The resulting habitat suitability surfaces were classified into five ordinal categories. This five-class scheme was adopted to provide a practical balance between model tractability for resistance analysis and ecological interpretability [16,19]. The categories allow for clear ecological interpretation, distinguishing habitat quality across five distinct levels: Very High Suitability (Score 5), High Suitability (Score 4), Moderate Suitability (Score 3), Low Suitability (Score 2), and Very Low Suitability/Unsuitable (Score 1). For subsequent analyses, values ≥ 4 were interpreted as indicative of suitable habitat, enabling clear delineation of high-priority areas for connectivity and conservation analyses.

### 2.7. Landscape Connectivity

Circuit theory-based modeling and spatially explicit interpolation analyses were performed to assess the influence of spatial separation and landscape resistance on genetic structure. Pairwise resistance distances among sampling sites were calculated using Circuitscape version 4.0.5 [17] in the pairwise-modeling mode, with resistance surfaces derived from MaxEnt-based habitat suitability maps. To construct these resistance surfaces, we applied a negative linear transformation to convert habitat suitability indices into resistance values (Resistance = 1 − Suitability). This linear approach was chosen as a conservative assumption following standard landscape genetics practices [18], as the specific physiological costs of movement for elephants in this complex landscape have not yet been empirically quantified. Current flow maps were used to represent cumulative movement costs across the landscape, enabling biological interpretation of movement corridors and barriers. For each environmental variable, a Mantel test was performed using the mantel () function in the vegan package for R [52] for assessing the correlation between genetic and resistance distances, identifying landscape features most strongly associated with genetic structure. The analysis was performed separately for each study site (PK and ARN), and the resulting resistance distances were compared with mitochondrial genetic distances using Mantel tests implemented via the mantel () function in the vegan package [52] in R for assessing IBR. IBD was independently assessed using Alleles in Space (AIS) version 1.0 [53], in which Mantel tests were conducted to evaluate the correlation between pairwise genetic and geographic distances among samples within each population. A complementary perspective on spatial genetic structure, which is independent of landscape resistance, was provided, allowing direct comparison of IBR and IBD patterns. To further visualize the fine-scale spatial patterns of genetic differentiation, landscape shape interpolation (LSI) was conducted in AIS for both populations. Raw mitochondrial genetic distances were used as surface heights (Z), and universal transverse mercator coordinates were assigned to the X and Y dimensions. Surface interpolation was performed using inverse distance weighting with a distance weighting parameter (α) = 2. The resulting three-dimensional surfaces highlighted spatial peaks corresponding to regions with elevated genetic dissimilarity (potential barriers) and troughs indicating genetically homogeneous regions (potential corridors). Grid resolution was automatically set by AIS. In LSI analysis, spatial peaks in the interpolated genetic surface were interpreted as areas where elevated genetic dissimilarities, which are potential barriers to gene flow, were present, whereas troughs indicated regions characterized by genetic homogeneity, which are potential corridors. The spatial autocorrelation index (V) was calculated in AIS for each site to assess spatial autocorrelation. Distance classes constructed using unequal distances with equal sample sizes were used, and the significance of the observed spatial autocorrelation was evaluated by permutation testing, in which 1000 permutations were performed. *p*-values < 0.05 were considered significant. This method helped in identifying spatial clustering or structure in genetic data, which is beyond that expected under spatial randomness, following the criteria used in other spatial genetic analyses

## 3. Results

### 3.1. Genetic Diversity, Population Structure, and Demographic Scenario

**Figure 1:**
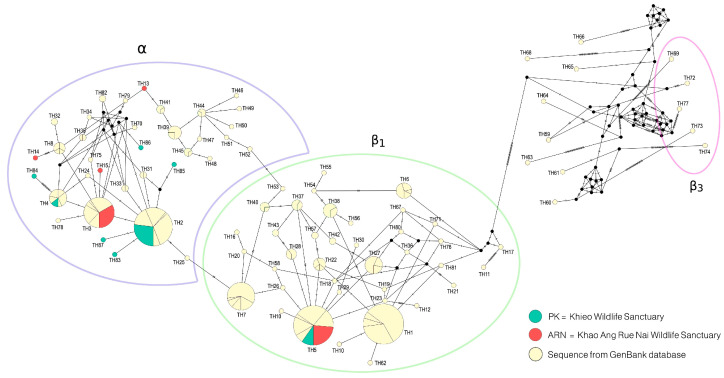
Median-joining haplotype network based on mtDNA D-loop sequences of Thai elephants. Circle sizes correspond to haplotype frequencies; colors represent sampling sites.

**Figure 2:**
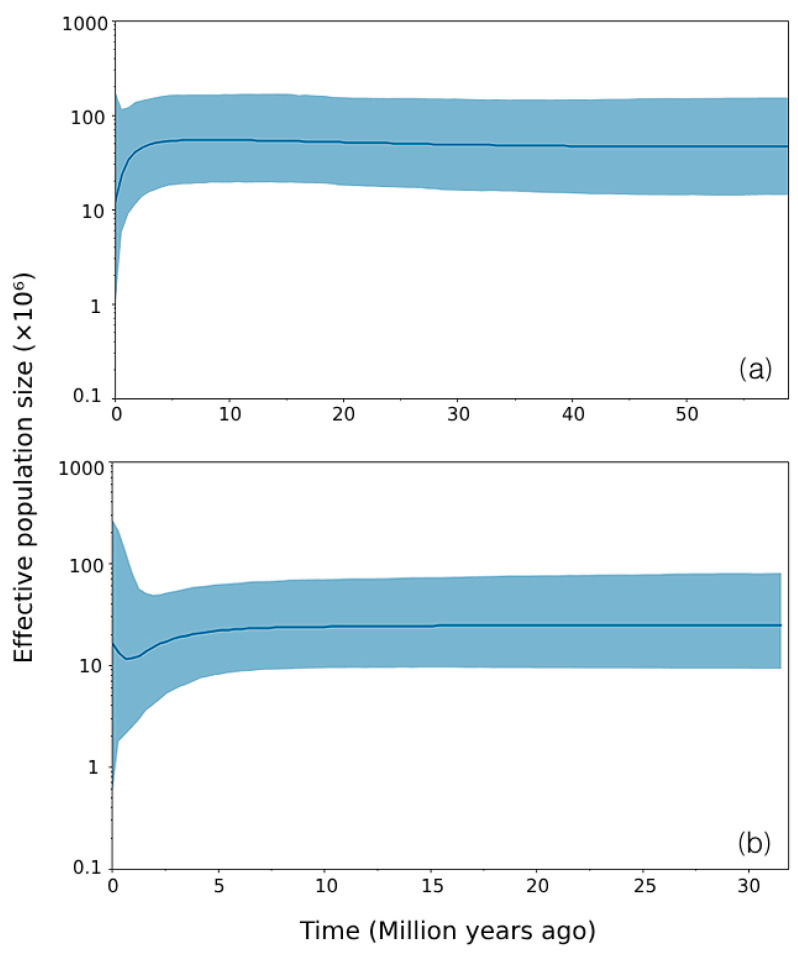
Bayesian skyline plot for maternal lineages of Thai elephants in this study (**a**) Phu Khieo Wildlife Sanctuary (PK); (**b**) Khao Ang Rue Nai Wildlife Sanctuary (ARN).

A total of 66 mtDNA D-loop sequences, including 32 from PK and 34 from ARN, were aligned to 359 bps, revealing 52 polymorphic sites and 14 haplotypes. In PK, *h* was 0.712  ±  0.005; *π* was 0.020  ±  0.000; and the mean number of nucleotide differences was 7.012. In ARN, *h* was 0.624  ±  0.002; *π* was 0.022  ±  0.000; and the average number of nucleotide differences was 7.914 (Table 1). Only haplotype TH2 was shared between populations; all other haplotypes were population-specific (Table S3). AMOVA showed that 11.65% of the total genetic variation occurred among populations, and 88.35% occurred within populations (Table S4). *G*_ST_ was 0.1601, and both Φ_ST_ and *F*_ST_ were 0.1165 and were statistically significant (*F*_ST_, *p* < 0.0001; Φ_ST_, *p* = 0.0068). *D*_xy_ was 0.0237, and *D*_a_ was 0.0028; *N*_m_ was 1.89. A median-joining haplotype network (Figure 1) revealed 87 unique haplotypes classified into three major haplogroups: α, β1, and β3. Haplotypes from PK and ARN were observed in both α and β_1_ haplogroups, with only one haplotype (TH5) shared between the two populations. In the α clade, the most common haplotypes were TH2 and TH3, while in the β_1_ clade, the most common haplotypes were TH1 and TH5. The β3 haplogroup, which was a densely interconnected cluster composed entirely of mtDNA D-loop sequences, contained no haplotypes from ARN or PK, and was solely from the elephant population of the National Elephant Institute [11]. The maximum likelihood phylogenetic tree, which revealed three major clades corresponding to α, β_1_, and β_3_, was consistent with the groupings observed in the haplotype network (Figure S3). The α clade, which contained 25 PK and 17 ARN samples and the β1 clade, which included 7 PK and 17 ARN samples, are depicted in the tree. Both PK and ARN were absent in the β3 clade. Neutrality tests yielded nonsignificant results for both populations (Table 2). In PK, Tajima’s *D* was −1.366, Fu and Li’s D* was −2.291, Fu and Li’s F* was −2.345, and Fu’s *Fs* was 2.906. In ARN, Tajima’s D was 1.025, Fu and Li’s D* was −1.003, Fu and Li’s F* was −0.396, and Fu’s *Fs* was 7.911. Mismatch distribution analyses revealed contrasting demographic histories between the populations (Table S5). The PK population exhibited a unimodal distribution with low SSD (0.085, *p* = 0.130) and raggedness index (0.175, *p* = 0.060). Conversely, the ARN population showed a multimodal distribution with a relatively high SSD (0.229, *p* < 0.05) and raggedness index (0.425, *p* = 0.080) (Figure S2). An exploratory BSP analysis was conducted to provide a visual representation of these demographic patterns over time. The analysis revealed that *N*_e_ in PK remained stable over time, with a slight recent decline, whereas *N*_e_ in ARN also remained stable (Figure 2). These results, based on mtDNA haplotype counts from non-invasive samples, provide valuable insights into maternal lineage history, though the exact number of unique individuals remains uncertain. Wide BSP credible intervals indicate substantial uncertainty; consequently, these results are treated as exploratory.

### 3.2. Habitat Suitability and Landscape Resistance

Habitat suitability models were independently developed for PK and ARN using the MaxEnt algorithm based on presence-only occurrence records and 12 environmental variables. Both models demonstrated high discriminatory performance, with AUC values of 0.994 for PK and 0.996 for ARN (Table S6), indicating robust predictive capacities. Jackknife analyses of regularized training gain identified slope, TRI, and canopy height as the most influential predictors in the PK model. By contrast, the ARN model was most strongly influenced by anthropogenic features, particularly the degree of urbanization and distance to major roads (Figure S4). Several variables, including elevation, percentage of tree cover, and forest type, were consistently informative across both models, indicating shared environmental determinants of habitat suitability. Habitat suitability classifications based on MaxEnt probability scores (class ≥ 4) were used to delineate zones of suitable and unsuitable habitat across the two study sites (Figure S5). Suitable habitats in PK were distributed in large and contiguous patches, primarily concentrated in the central and northern regions of the sanctuary. Comparatively, suitable areas in ARN were relatively highly fragmented and mainly located in the central and northeastern regions. The habitat suitability outputs were subsequently transformed into resistance surfaces using a negative exponential function, which assigned low resistance values to high-suitability areas. These resistance layers were used in subsequent connectivity and gene flow analyses. In both sites, areas with high suitability corresponded to low resistance values, whereas regions affected by anthropogenic disturbances, such as roads, settlements, and rugged terrain, were represented by high resistance values, indicating limited permeability to movement.

### 3.3. Landscape Connectivity and Isolation by Distance

**Figure 3:**
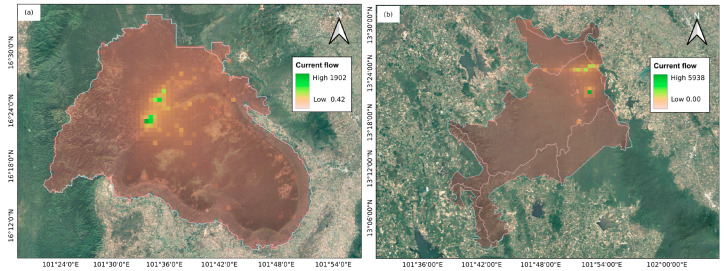
Circuitscape resistance surface for species connectivity. (**a**) Phu Khieo Wildlife Sanctuary (PK); (**b**) Khao Ang Rue Nai Wildlife Sanctuary (ARN).

**Figure 4:**
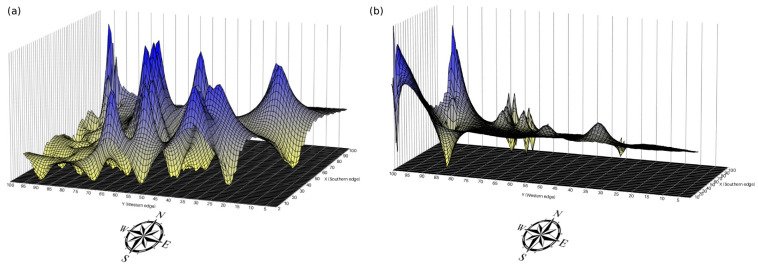
Interpolated genetic landscape surfaces for (**a**) Phu Khieo Wildlife Sanctuary (PK) and (**b**) Khao Ang Rue Nai Wildlife Sanctuary (ARN).

A series of analyses were performed using the mtDNA data to assess the influence of landscape features on maternal-lineage connectivity. Circuit-based resistance surfaces were generated for both study sites (Figure 3). In PK, low-resistance zones were observed within the central forest core, with resistance increasing outward toward the sanctuary boundaries. By contrast, ARN exhibited low resistance in the northern regions, with high-flow areas observed near disturbed regions and road networks. Genetic and environmental correlations assessed using Mantel tests showed that in PK, significant associations were detected between mtDNA genetic distance and distance to major roads, TWI, and distance to streams (Table S7), whereas the composite resistance model did not show significant correlations with genetic distance. In ARN, significant positive correlations were observed with the degree of urbanization, distance to major roads, and nighttime light. These results indicate distinct associations between landscape features and mtDNA genetic distance in each population, reflecting differences in resistance surface patterns. Despite these associations, no evidence of IBR was found in either site for maternal lineages, as the correlations between resistance and mtDNA genetic distances were low and not significant (PK: *r* = −0.102, *p* = 0.769; ARN: *r* = 0.046, *p* = 0.135) (Figure S6). Similarly, IBD was not supported, with weak and nonsignificant correlations observed between mtDNA genetic and geographic distances (PK: *r* = 0.057, *p* = 0.270; ARN: *r* = 0.058, *p* = 0.168) (Figure S7). The LSI maps generated via AIS revealed spatial variations in maternal-lineage genetic distance across the study area (Figure 4). In PK, the LSI surface, which exhibited multiple peaks particularly along the edges of the central forest core, indicated areas of relatively high mtDNA genetic distance and relatively strong spatial genetic structure among samples at the forest margins, whereas the central core remained relatively flat, suggesting potential corridors. In ARN, the LSI surface, which was relatively flat with only a few peaks mostly in the southern and western disturbed areas, reflected an overall low spatial maternal-lineage genetic structure. V for PK was significant (V = 0.00793, *p* < 0.01), indicating a significant positive autocorrelation at the distance classes of 3528–7444 and 19,007–21,453 m. By contrast, overall V for ARN was not significant (V = 0.02902, *p* = 0.999), showing that none of the 10 distance classes exhibited significant autocorrelation (all *p* > 0.05) (Table S7).

## 4. Discussion

In this study, habitat suitability in PK and ARN was assessed to test whether habitat fragmentation and development are associated with relatively high landscape resistance in ARN. MaxEnt models demonstrated high predictive performance and identified natural variables, such as slope, TRI, and canopy height, as key predictors. Elephants primarily utilize the forest interior for resources [26]. MaxEnt models showed excellent predictive capacity for ARN, a lowland landscape with approximately 30% of its original forest cover remaining after decades of agricultural and infrastructure development [27]. Anthropogenic variables, such as urbanization and proximity to roads, were the strongest negative predictors of elephant occurrence. Suitable habitats were fragmented and were mainly located in the northeastern sectors, particularly near road networks. Despite classification as low suitability, elephants frequently utilize croplands, orchards, and roadside vegetation, probably owing to limited natural forage in more suitable habitats [23,27].

The habitat suitability pattern, which aligns with the maternal genetic structure in both PK and ARN populations, showed that higher *h* value observed in PK than in ARN, reflected recent population changes, while similar *π* values indicated comparable genetic variation [54]. Population demographic analyses, including neutrality tests and unimodal mismatch distributions with a low and nonsignificant raggedness index (except for BSP results, interpreted cautiously due to wide confidence intervals), suggested that the PK population had undergone expansion after a recent bottleneck. The presence of large, contiguous, and suitable patches in PK supports population survival and growth, whereas relatively highly fragmented and isolated areas in ARN may pose challenges to dispersal. This may also reflect the stability of the Asian elephant population in ARN, as depicted by genetic results. Most samples in PK, which were found mainly in the α clade, reflected a potentially matrilineal homogeneity, while samples in ARN, which were distributed across both α and β1 clades, indicated relatively broad retention of maternal lineages. This pattern may result from differences in historical connectivity, local lineage persistence, recent demographic processes, or habitat structure in both PK and ARN [11,13,25]. Notably, historical records suggesting that elephants moved freely across central and eastern Thailand until the late 19th century [55] indicate that forest connectivity between PK and ARN likely persisted initially. However, increasing anthropogenic pressures, such as illegal logging and agricultural expansion, have likely associated with disrupted connectivity, contributing to the fragmentation at present [56,57]. Genetic differentiation and AMOVA results, which indicated limited maternal lineage exchange between the PK and ARN populations, suggested that landscape and habitat influenced the genetic connectivity between the two elephant populations. Only one haplotype (TH5) was shared, with the remainder being population-specific, suggesting that fragmented and isolated areas were likely associated with the movement and expansion of the Asian elephant population. Our sample-based mtDNA analysis reflects only maternal lineages, providing insight into maternal-lineage connectivity and historical patterns.

In both PK and ARN, resistance surfaces identified landscape features likely to impede elephant movement. In PK, significant autocorrelation was limited to specific distance classes, indicating fine-scale genetic structuring that was not captured by the overall linear trends in IBD or IBR analyses [58]. This suggests that spatial genetic structures may occur at discrete scales because of localized processes or behaviors, particularly in heterogeneous or human-modified landscapes. Localized peaks of genetic dissimilarity in ARN, specifically near the disturbed southern sectors that corresponded to high-resistance areas, were identified. In PK, relatively smooth genetic surfaces with elevated values near sanctuary boundaries probably reflect edge effects or bottlenecks in local movement [17]. Although landscape resistance models are widely used to predict genetic structures in fragmented populations, such models alone may not always accurately capture female-mediated connectivity, specifically in highly mobile and adaptable species, such as elephants. Factors that may explain the lack of a clear link between the modeled resistance and observed genetic structure include the undisturbed landscape in PK, which probably allows for unrestricted movement and connectivity, aligning with local genetic structuring. In ARN, behavioral adaptability may enable elephants to cross or utilize high-resistance areas, overriding model predictions.

A primary limitation of this study stems from the reliance on mtDNA D-loop sequences, which were amplified from non-invasively collected fecal samples. While fecal sampling is essential for monitoring endangered megafauna, the resulting templates often suffer from significant DNA degradation and low endogenous concentrations, which present substantial technical hurdles for the consistent recovery of the high-quality nuclear genomic markers required for individual identification. Because mtDNA exists in significantly higher copy numbers per cell than nuclear DNA, it remains more accessible in environmental samples; however, this dependence on matrilineal inheritance captures only haplotype distributions rather than true nuclear allele frequencies. This distinction is critical, as it restricts the ability to distinguish between individuals or to estimate fine-scale demographic parameters, such as effective population size and male-mediated gene flow, which cannot be robustly quantified without biparental genomic data. It is also important to consider the temporal scale mismatch between historical mtDNA markers and recent landscape changes; therefore, our results should be interpreted as correlational rather than causal. These findings reflect associations with contemporary landscape features that may represent disruption of historical migration routes or patterns of female philopatry, rather than de novo genetic divergence driven solely by recent infrastructure. Furthermore, the spatial clustering of samples and the relatively modest sample size represent constraints that may limit the broader representativeness of the dataset and influence the sensitivity of the circuit-based connectivity models. Because resistance surfaces were derived from occurrence-based habitat suitability models, potential circularity should be considered, and resistance analyses are treated as exploratory. Additionally, although distinct dung piles were selected based on physical characteristics (e.g., bolus size and decay stage), non-invasive sampling at 1-m intervals may risk repeated sampling; thus, these landscape genetic models should be interpreted with caution. While this integrated approach provides preliminary insights into maternal lineage structure and historical isolation, genome-wide markers remain indispensable for verifying individual identities and for validating the landscape-genetic inferences that are central to long-term population viability assessments.

Beyond Thailand, these findings provide important lessons for Asian elephant conservation in other range countries facing rapid habitat fragmentation. The contrast between PK (continuous forest) and ARN (highly fragmented) highlights the urgency of maintaining connectivity in lowland populations. Maternal lineage data reveal localized genetic isolation, emphasizing the need for integrated monitoring that combines genetic, ecological, and behavioral information to detect fragmentation early. Where resources for large-scale genomic studies are limited, mtDNA haplotype analysis with Circuit Theory offers a practical first-phase tool to identify “pinch points”, defined as areas of elevated resistance and isolation risk [59]. Targeted interventions such as wildlife crossings, ecological corridors, and community-based land-use planning are critical to sustain connectivity and secure elephant populations across Southeast Asia. Our recommendations for implementation, which have policy implications, can be addressed through a policy laboratory. In PK, the landscape, which remains relatively continuous with intact forests and minimal barriers, supports broad elephant movement and genetic connectivity. Although barriers, such as major roads, exist; overall dispersal is effective, resulting in low genetic differentiation. Protecting habitat corridors and satellite monitoring of land-use changes are essential for preserving connectivity in PK [60]. By contrast, in ARN, barriers, such as roads, agriculture, and settlements, are associated with maternal lineage structuring inferred from mtDNA, suggesting reduced female-mediated connectivity rather than direct movement restriction [61,62,63]. These features increase genetic differentiation and potential inbreeding risks, emphasizing the urgent need for conservation to restore connectivity. To address habitat fragmentation in ARN, efforts should be made to establish and protect ecological corridors connecting the remaining forest patches, as previously recommended [64]. Infrastructure planning, specifically along Highway 3076 (13°25′1.21″ N, 101°52′49.74″ E) where elephant movement is frequent, should incorporate wildlife overpasses or underpasses for reducing barrier effects, as previously suggested [65,66]. In agricultural landscapes, community-based land-use planning and incentives for buffer zones with elephant-friendly crops can minimize conflict and support connectivity [67]. Species-specific responses to fragmentation [16,61], should be understood to ensure long-term genetic exchange, population viability, and resilience amid ongoing human pressure [68].

## 5. Conclusions

This study highlights that, while large-scale resistance models provide a useful ecological context, patterns of gene flow and genetic variation in Asian elephants are intricately influenced by local landscape features, behavior, and specific land-use dynamics. The inherent limitations of mtDNA in capturing recent or paternal gene flow suggest that genome-wide nuclear data, which offer significantly higher resolution and individual identification, should be incorporated into future studies. This need for high resolution is critical, as it refers not only to the genetic data but also to the fine-scale (~30-m) raster layers used in our habitat and resistance modeling, which are vital for spatial accuracy. However, a recognized trade-off exists between achieving finer spatial resolution and maintaining model reliability for broad movement inference [69]. Additionally, future connectivity models should integrate ecological resistance surfaces with empirical behavioral data to accurately represent landscape permeability and its impact on gene flow. A key methodological limitation of this study is the reliance on mtDNA D-loop data from non-invasive fecal samples, which reflects only maternal haplotype frequencies rather than true nuclear allele frequencies and precludes reliable estimation of the number of individuals sampled. Consequently, robust population-level inferences on contemporary gene flow and demography remain constrained without comprehensive nuclear genomic data. In addition, the relatively modest and spatially clustered samples represent a limitation that may reduce the robustness of the circuit-based connectivity analyses presented here.

## Figures and Tables

**Table 1:** Genetic diversity indices for mtDNA D-loop sequences in wild Asian elephants in Thailand in this study.

Population	Sample Size	Number of Haplotypes (H)	Theta (Per Site) from S	Average Number of Nucleotide Differences (k)	Haplotype Diversity (h)	Nucleotide Diversities (π)
PK ^1^	32	9	0.031	7.012	0.712 ± 0.005	0.020 ± 0.000
ARN ^2^	34	6	0.017	7.914	0.624 ± 0.002	0.022 ± 0.000
Total	66	14	0.033	7.983	0.783 ± 0.001	0.022 ± 0.000

^1^ PK = Phu Khieo Wildlife Sanctuary, Chaiyaphum; ^2^ ARN = Khao Ang Rue Nai Wildlife Sanctuary, Chachoengsao.

**Table 2:** Neutrality tests of mtDNA D-loop sequences in wild Asian elephants in Thailand.

Population	Tajima	Fu D*	Fu F*	Fu’s *F_s_*
PK ^1^	−1.366 ^ns^	−2.291 ^ns^	−2.345 ^ns^	2.906
ARN ^2^	1.025 ^ns^	−1.003 ^ns^	−0.396 ^ns^	7.911
Total	−1.078 ^ns^	−4.215 *	−3.602 *	2.603

^ns^ = not significant; * Significant at *p* < 0.05; ^1^ PK = Phu Khieo Wildlife Sanctuary, Chaiyaphum; ^2^ ARN = Khao Ang Rue Nai Wildlife Sanctuary, Chachoengsao.

## Data Availability

The 66 mtDNA D-loop sequences from wild elephants have been deposited in GenBank (accession numbers PV649948–PV650013).

## Supplementary Materials

The following supporting information can be downloaded at: https://www.mdpi.com/article/10.3390/biology15040358/s1, Figure S1: Geographic locations of the two study areas in Thailand. Figure S2: Mismatch distribution of pairwise nucleotide differences under the sudden expansion model. Figure S3: Maximum Likelihood (ML) of mitochondrial DNA D-loop haplotypes from elephants in PK and ARN. Branch lengths represent relative divergence times. Figure S4: Jackknife of regularized training gain for environmental variables in species modeling. Figure S5: Habitat suitability classification for Asian elephant. Figure S6: Relationship between resistance distance and genetic distance (Isolation by Resistance) in Asian elephants. Figure S7: Relationship between geographic distance and genetic distance (Isolation by Distance) in Asian elephants. Table S1: Summary of mitochondrial DNA D-loop sequences used in this study. Table S2: Spatial variables used to construct resistance layers for landscape resistance modeling. Table S3: Haplotype distribution of mitochondrial DNA D-loop sequences in wild Asian elephants from PK and ARN Wildlife Sanctuaries. Table S4: AMOVA results for mitochondrial DNA D-loop sequences from wild Asian elephants in Thailand. Table S5: Mismatch distribution parameters and demographic expansion test results for mitochondrial DNA sequences in PK and ARN populations. Table S6: Mantel test results showing correlations (*r*) and *p*-values between genetic distance and environmental resistance variables for PK and ARN. Table S7: Results of spatial autocorrelation analyses (LSI) for wild Asian elephants in PK and ARN. References [11,25,70,71,72,73,74,75,76,77,78,79,80,81,82] are cited in the supplementary materials.

## Abbreviations

The following abbreviations are used in this manuscript:

PK	Phu Khieo Wildlife Sanctuary
ARN	Khao Ang Rue Nai Wildlife Sanctuary

## References

[1] Fahrig L. (2003). Effects of habitat fragmentation on biodiversity. Annu. Rev. Ecol. Evol. Syst..

[2] Haddad N.M., Brudvig L.A., Clobert J., Davies K.F., Gonzalez A., Holt R.D., Lovejoy T.E., Sexton J.O., Austin M.P., Collins C.D. (2015). Habitat fragmentation and its lasting impact on Earth’s ecosystems. Sci. Adv..

[3] Frankham R. (2005). Genetics and extinction. Biol. Conserv..

[4] Lande R. (1988). Genetics and demography in biological conservation. Science.

[5] Williams C., Tiwari S.K., Goswami V.R., de Silva S., Kumar A., Baskaran N., Yoganand K., Menon V. *Elephas maximus*. The IUCN Red List of Threatened Species 2020: E.T7140A45818198. https://www.iucnredlist.org/species/7140/45818198.

[6] Menon V., Tiwari S. (2019). Population status of Asian elephants *Elephas maximus* and key threats. Int. Zoo Yearb..

[7] Department of National Parks, Wildlife and Plant Conservation (2025). Wild Elephant Population in Conservation Areas, Fiscal Year 2025.

[8] Ariyaraphong N., Laopichienpong N., Singchat W., Panthum T., Ahmad S.F., Jattawa D., Duengkae P., Muangmai N., Suwanasopee T., Koonawootrittriron S. (2021). High-level gene flow restricts genetic differentiation in dairy cattle populations in Thailand: Insights from large-scale mt D-loop sequencing. Animals.

[9] Pavlova A., Beheregaray L.B., Coleman R., Gilligan D., Harrisson K.A., Ingram B.A., Kearns J., Lamb A.M., Lintermans M., Lyon J. (2017). Severe consequences of habitat fragmentation on genetic diversity of an endangered Australian freshwater fish: A call for assisted gene flow. Evol. Appl..

[10] Wattanadilokchatkun P., Chaiyes A., Ariyaraphong N., Wongloet W., Suksavate W., Thatukan C., Kumnan N., Panthum T., Thong T., Singchat W. (2024). Integrative approach for landscape demography analysis of Plakad-Pa Pak-Tawan-Ok (*Betta siamorientalis*): Deciphering genetic and environmental factors in Eastern Thailand’s conservation efforts. Glob. Ecol. Conserv..

[11] Ariyaraphong N., Ho My Nguyen D., Singchat W., Suksavate W., Panthum T., Langkaphin W., Chansitthiwet S., Angkawanish T., Promking A., Kaewtip K. (2022). Standard identification certificate for legal legislation of a unique gene pool of Thai domestic elephants originating from a male elephant contribution to breeding. Sustainability.

[12] Jangtarwan K., Kamsongkram P., Subpayakom N., Sillapaprayoon S., Muangmai N., Kongphoemph A., Wongsodchuen A., Intapan S., Chamchumroon W., Safoowong M. (2020). Predictive genetic plan for a captive population of the Chinese goral (*Naemorhedus griseus*) and prescriptive action for ex situ and in situ conservation management in Thailand. PLoS ONE.

[13] Srikulnath K., Ariyaraphong N., Singchat W., Panthum T., Lisachov A., Ahmad S.F., Han K., Muangmai N., Duengkae P. (2022). Asian elephant evolutionary relationships: New perspectives from mitochondrial D-loop haplotype diversity. Sustainability.

[14] van Strien M.J., Grêt-Regamey A. (2016). How is habitat connectivity affected by settlement and road network configurations? Results from simulating coupled habitat and human networks. Ecol. Model..

[15] Chibeya D., Wood H., Cousins S., Carter K., Nyirenda M.A., Maseka H. (2021). How do African elephants utilize the landscape during wet season? A habitat connectivity analysis for Sioma Ngwezi landscape in Zambia. Ecol. Evol..

[16] Zeller K.A., McGarigal K., Whiteley A.R. (2012). Estimating landscape resistance to movement: A review. Landscape Ecol..

[17] Dickson B.G., Albano C.M., Anantharaman R., Beier P., Fargione J., Graves T.A., Gray M.E., Hall K.R., Lawler J.J., Leonard P.B. (2019). Circuit-theory applications to connectivity science and conservation. Conserv. Biol..

[18] McRae B.H., Dickson B.G., Keitt T.H., Shah V.B. (2008). Using circuit theory to model connectivity in ecology, evolution, and conservation. Ecology.

[19] Pelletier D., Clark M., Anderson M.G., Rayfield B., Wulder M.A., Cardille J.A. (2014). Applying circuit theory for corridor expansion and management at regional scales: Tiling, pinch points, and omnidirectional connectivity. PLoS ONE.

[20] Fernando P., Pfrender M.E., Encalada S.E., Lande R. (2000). Mitochondrial DNA variation, phylogeography and population structure of the Asian elephant. Heredity.

[21] Storfer A., Murphy M.A., Evans J.S., Goldberg C.S., Robinson S., Spear S.F., Dezzani R., Delmelle E., Vierling L., Waits L.P. (2007). Putting the ‘landscape’ in landscape genetics. Heredity.

[22] Khan A., Sil M., Thekaekara T., Garg K.M., Sinha I., Khurana R., Sukumar R., Ramakrishnan U. (2024). Divergence and serial colonization shape genetic variation and define conservation units in Asian elephants. Curr. Biol..

[23] Sinovas P., Smith C., Keath S., Chantha N., Kaden J., Ith S., Ball A. (2025). Giants in the landscape: Status, genetic diversity, habitat suitability and conservation implications for a fragmented Asian elephant (*Elephas maximus*) population in Cambodia. PeerJ.

[24] Keller I., Largiadèr C.R. (2003). Recent habitat fragmentation caused by major roads leads to reduction of gene flow and loss of genetic variability in ground beetles. Proc. R. Soc. Lond. B.

[25] Quainoo D.K., Chalermwong P., Muangsuk P., Nguyen T.H., Panthum T., Singchat W., Budi T., Duengkae P., Suksavate W., Chaiyes A. (2025). Genetic insights for enhancing conservation strategies in captive and wild Asian elephants through improved non-invasive DNA-based individual identification. PLoS ONE.

[26] Htet N.N.P., Chaiyarat R., Thongthip N., Anuracpreeda P., Youngpoy N., Chompoopong P. (2021). Population and distribution of wild Asian elephants (*Elephas maximus*) in Phu Khieo Wildlife Sanctuary, Thailand. PeerJ.

[27] Menkham K., Sukmasuang R., Pla-Ard M., Charaspet K., Panganta T., Trisurat Y., Bhumpakphan N. (2019). Population and habitat use of Asian elephants (*Elephas maximus*) and five ungulate species in Khao Ang Rue Nai wildlife sanctuary, Chachoengsao Province, Thailand. Biodiversitas.

[28] Kitratporn N., Takeuchi W. (2019). Spatiotemporal distribution of human–elephant conflict in eastern Thailand: A model-based assessment using news reports and remotely sensed data. Remote Sens..

[29] International Centre for Environmental Management (2003). Thailand National Report on Protected Areas and Development.

[30] Sukmasuang R., Phumpakphan N., Deungkae P., Chaiyarat R., Pla-Ard M., Khiowsree N., Charaspet K., Paansri P., Noowong J. (2024). Review: Status of wild elephant, conflict and conservation actions in Thailand. Biodiversitas.

[31] Fernando P., Vidya T.N., Rajapakse C., Dangolla A., Melnick D.J. (2003). Reliable noninvasive genotyping: Fantasy or reality?. J. Hered..

[32] Tamura K., Stecher G., Kumar S. (2021). MEGA11: Molecular evolutionary genetics analysis version 11. Mol. Biol. Evol..

[33] Rozas J., Ferrer-Mata A., Sánchez-DelBarrio J.C., Guirao-Rico S., Librado P., Ramos-Onsins S.E., Sánchez-Gracia A. (2017). DnaSP 6: DNA sequence polymorphism analysis of large data sets. Mol. Biol. Evol..

[34] Excoffier L., Lischer H.E. (2010). Arlequin suite ver 3.5: A new series of programs to perform population genetics analyses under Linux and Windows. Mol. Ecol. Resour..

[35] Weir B.S., Cockerham C.C. (1984). Estimating F-statistics for the analysis of population structure. Evolution.

[36] Excoffier L., Smouse P.E., Quattro J.M. (1992). Analysis of molecular variance inferred from metric distances among DNA haplotypes: Application to human mitochondrial DNA restriction data. Genetics.

[37] Wright S. (1978). The relation of livestock breeding to theories of evolution. J. Anim. Sci..

[38] Leigh J.W., Bryant D., Nakagawa S. (2015). POPART: Full-feature software for haplotype network construction. Methods Ecol. Evol..

[39] Nguyen L.T., Schmidt H.A., Von Haeseler A., Minh B.Q. (2015). IQ-TREE: A fast and effective stochastic algorithm for estimating maximum-likelihood phylogenies. Mol. Biol. Evol..

[40] Kalyaanamoorthy S., Minh B.Q., Wong T.K.F., Von Haeseler A., Jermiin L.S. (2017). ModelFinder: Fast model selection for accurate phylogenetic estimates. Nat. Methods.

[41] Letunic I., Bork P. (2007). Interactive Tree Of Life (iTOL): An online tool for phylogenetic tree display and annotation. Bioinformatics.

[42] Rogers A.R., Harpending H. (1992). Population growth makes waves in the distribution of pairwise genetic differences. Mol. Biol. Evol..

[43] Suchard M.A., Lemey P., Baele G., Ayres D.L., Drummond A.J., Rambaut A. (2018). Bayesian phylogenetic and phylodynamic data integration using BEAST 1.10. Virus Evol..

[44] Drummond A.J., Suchard M.A., Xie D., Rambaut A. (2012). Bayesian phylogenetics with BEAUti and the BEAST 1.7. Mol. Biol. Evol..

[45] Palkopoulou E., Lipson M., Mallick S., Nielsen S., Rohland N., Baleka S., Karpinski E., Ivancevic A.M., To T.H., Kortschak R.D. (2018). A comprehensive genomic history of extinct and living elephants. Proc. Natl. Acad. Sci. USA.

[46] Rambaut A., Drummond A.J., Xie D., Baele G., Suchard M.A. (2018). Posterior summarization in Bayesian phylogenetics using Tracer 1.7. Syst. Biol..

[47] Phillips S.J., Anderson R.P., Schapire R.E. (2006). Maximum entropy modeling of species geographic distributions. Ecol. Model..

[48] Phillips S.J., Dudík M. (2008). Modeling of species distributions with Maxent: New extensions and a comprehensive evaluation. Ecography.

[49] Shi M., Tian Y., Tang Y., Yang K., Zhang L., Zhang S., Tang R., Bao M., Sun G. (2025). Population genetics reveal potential threats from low maternal genetic diversity in wild Asian elephants in China. Glob. Ecol. Conserv..

[50] QGIS Development Team (2025). QGIS Geographic Information System (Version 3.40). QGIS Association. https://www.qgis.org.

[51] Jiménez-Valverde A. (2012). Insights into the area under the receiver operating characteristic curve (AUC) as a discrimination measure in species distribution modelling. Glob. Ecol. Biogeogr..

[52] Dixon P. (2003). VEGAN, a package of R functions for community ecology. J. Veg. Sci..

[53] Miller M.P. (2005). Alleles In Space (AIS): Computer software for the joint analysis of interindividual spatial and genetic information. J. Hered..

[54] Grant W.A.S., Bowen B.W. (1998). Shallow population histories in deep evolutionary lineages of marine fishes: Insights from sardines and anchovies and lessons for conservation. J. Hered..

[55] Chularat C. (2004). Elephant as a commodity: Elephant trade in Ayutthaya period. Silpa-mag..

[56] Leimgruber P., Gagnon J.B., Wemmer C., Kelly D.S., Songer M.A., Selig E.R. (2003). Fragmentation of Asia’s remaining wildlands: Implications for Asian elephant conservation. Anim. Conserv..

[57] Bhumpakphan N., Sukmasuang R., Thongtip N., Chimchome V., Trisurat Y., Yindee M. (2013). Strategic plan for elephant conservation and mitigation of their problems in Thailand. Proceedings of the 34th Thailand Wildlife Seminar, Bangkok, Thailand, 19–20 December 2013.

[58] Diniz-Filho J.A.F., Soares T.N., Lima J.S., Dobrovolski R., Landeiro V.L., Telles M.P., Rangel T.F., Bini L.M. (2013). Mantel test in population genetics. Genet. Mol. Biol..

[59] Cushman S.A., McRae B., Adriaensen F., Beier P., Shirley M., Zeller K., Macdonald D.W., Willis K.J. (2013). Biological corridors and connectivity. Key Topics in Conservation Biology 2.

[60] Suksavate W., Duengkae P., Chaiyes A. (2019). Quantifying landscape connectivity for wild Asian elephant populations among fragmented habitats in Thailand. Glob. Ecol. Conserv..

[61] Blake S., Deem S.L., Strindberg S., Maisels F., Momont L., Isia I.B., Douglas-Hamilton I., Karesh W.B., Kock M.D. (2008). Roadless wilderness area determines forest elephant movements in the Congo Basin. PLoS ONE.

[62] Laurance W.F. (2008). Theory meets reality: How habitat fragmentation research has transcended island biogeographic theory. Biol. Conserv..

[63] Wilson G., Gray R.J., Radinal R., Hasanuddin H., Azmi W., Sayuti A., Muhammad H., Abdullah A., Nazamuddin B.S., Sofyan H. (2021). Between a rock and a hard place: Rugged terrain features and human disturbance affect behaviour and habitat use of Sumatran elephants in Aceh, Sumatra, Indonesia. Biodivers. Conserv..

[64] Hilty J.A., Lidicker W.Z., Merenlender A.M. (2012). Corridor Ecology: The Science and Practice of Linking Landscapes for Biodiversity Conservation.

[65] Fahrig L., Rytwinski T. (2009). Effects of roads on animal abundance: An empirical review and synthesis. Ecol. Soc..

[66] van der Ree R., van der Grift E., Gulle N., Holland K., Mata C., Suarez F., Irwin C.L., Nelson D., McDermott K.P. (2007). Overcoming the barrier effect of roads—How effective are mitigation strategies? An international review of the use and effectiveness of underpasses and overpasses designed to increase the permeability of roads for wildlife. Proceedings of the 2007 International Conference on Ecology and Transportation, Raleigh, NC, USA, 14–18 May 2007.

[67] Sukumar R. (2003). The Living Elephants: Evolutionary Ecology, Behavior, and Conservation.

[68] Hodgson J.A., Thomas C.D., Wintle B.A., Moilanen A. (2009). Climate change, connectivity and conservation decision making: Back to basics. J. Appl. Ecol..

[69] Boyd R.J., Bowler D.E., Isaac N.J.B., Pescott O.L. (2024). On the trade-off between accuracy and spatial resolution when estimating species occupancy from geographically biased samples. Ecol. Model..

[70] Fickel J., Lieckfeldt D., Ratanakorn P., Pitra C. (2007). Distribution of haplotypes and microsatellite alleles among Asian elephants (*Elephas maximus*) in Thailand. Eur. J. Wildl. Res..

[71] Rutten G.P.A., Kumsuk M., Savini C., Pattanakaew P., Chareundong T., Manopawitr P., Kaewket C. Cytochrome b haplotypes of wild Asian elephant (*Elephas maximus*) populations in Kaeng Krachan National Park and Phukhieo Wildlife Sanctuary. Proceedings of the 29th Thailand Wildlife Seminar.

[72] Lei R., Brenneman R.A., Schmitt D.L., Louis E.E. (2011). Genetic diversity in North American captive Asian elephants. J. Zool..

[73] Meyer M., Palkopoulou E., Baleka S., Stiller M., Penkman K.E., Alt K.W., Hofreiter M. (2017). Palaeogenomes of Eurasian straight-tusked elephants challenge the current view of elephant evolution. eLife.

[74] Enk J., Devault A., Widga C., Saunders J., Szpak P., Southon J., Poinar H. (2016). Mammuthus Population Dynamics in Late Pleistocene North America: Divergence, Phylogeography, and Introgression. Front. Ecol. Evol..

[75] Kornienko I.V., Faleeva T.G., Oreshkova N.V., Grigoriev S.E., Grigoreva L.V., Simonov E.P., Krutovsky K.V. (2018). Complete mitochondrial genome of a woolly mammoth (*Mammuthus primigenius*) from Maly Lyakhovsky Island (New Siberian Islands, Russia) and its phylogenetic assessment. Mitochondrial DNA Part B.

[76] Debruyne R., Chu G., King C.E., Bos K., Kuch M., Schwarz C., Poinar H.N. (2008). Out of America: Ancient DNA Evidence for a New World Origin of Late Quaternary Woolly Mammoths. Curr. Biol..

[77] Lang N., Jetz W., Schindler K., Wegner J.D. (2023). A high-resolution canopy height model of the Earth. Nat. Ecol. Evol..

[78] Elvidge C.D., Zhizhin M., Ghosh T., Hsu F.C., Taneja J. (2021). Annual time series of global VIIRS nighttime lights derived from monthly averages: 2012 to 2019. Remote Sens..

[79] Farr T.G., Rosen P.A., Caro E., Crippen R., Duren R., Hensley S., Alsdorf D. (2007). The shuttle radar topography mission. Rev. Geophys..

[80] Gorelick N., Hancher M., Dixon M., Ilyushchenko S., Thau D., Moore R. (2017). Google Earth Engine: Planetary-scale geospatial analysis for everyone. Remote Sens. Environ..

[81] Schiavina M., Melchiorri M., Pesaresi M. (2023). GHS-SMOD R2023A-GHS Settlement Layers, Application of the Degree of Urbanisation Methodology (Stage I) to GHS-POP R2023A and GHS-BUILT-S R2023A, Multitemporal (1975–2030).

[82] WorldPop PopulationCounts2000–2020UN-Adjusted Unconstrained100m. 2020. [Data Set]. https://www.worldpop.org/.

