# Supplementary material for: Landscape Features Shape Maternal Genetic Structure of Asian Elephants in Thailand: Insights from mtDNA
Source: Biology (Basel). 2026 Feb 20;15(4):358. doi: 10.3390/biology15040358 (PMC12937948; doi:10.3390/biology15040358)
Supplement: Supplementary file 1 [file biology-15-00358-s001.zip › biology-4120614-supplementary.pdf]

Table S1. Summary of mitochondrial DNA D-loop sequences used in this study.

Source	Accession Numbers	Sample Origins	Usage in Study
This study	PV649948–PV650013	Fecal samples from wild elephants in Phu Khieo (PK) and Khao Ang Rue Nai (ARN), Thailand	Primary dataset, haplotype analysis, diversity, spatial genetics
Quainoo et al. (2025)	LC789761–LC789763, LC789922–LC789924	Captive elephants (EKS) and wild elephants in Thailand (Khao Yai, Khao Ang Rue Nai)	Haplotype clustering, phylogenetic comparison
Ariyaraphong et al. (2022)	LC700008–LC700047, LC699962–LC700007, LC699890–LC699961	Captive elephants in Thailand (BCEP, MEP, NEI)	Haplotype clustering, phylogenetic comparison
Fickel et al. (2007)	AF358446–AF358452 , AF317518–AF317535	Unknown locality in Thailand	Haplotype clustering, phylogenetic comparison
Dejchaisri et al. (2008)	KJ187772–KJ187802	Unknown locality in Thailand	Haplotype clustering, phylogenetic comparison
Lei et al. (2012)	FJ979436–FJ979629	Unknown locality in Thailand	Haplotype clustering, phylogenetic comparison
Meyer et al. (2017)	KY499555	*Elephas antiquus*	Phylogenetic outgroup
Enk et al. (2016)	KX027559	*Mammuthus jeffersonii*	Phylogenetic outgroup
Enk et al. (2016)	KX027513	*Mammuthus columbi*	Phylogenetic outgroup
Kornienko et al**.** (2018)	MF770243	*Mammuthus primigenius*	Phylogenetic outgroup
Enk et al. (2016)	KX027528	*Mammuthus sp.*	Phylogenetic outgroup
Debruyne (2005)	AY742802	*Loxodonta africana*	Phylogenetic outgroup

Table S2. Spatial variables used to construct resistance layers for landscape resistance modeling.

Category	Variable Name	Unit / Scale	Resolution	Notes	Source Description	Source
Natural	Canopy Height	meters	30 m	Dense forest = high resistance	Global Forest Canopy	Lang et al. (2023)
	Slope	degrees	30 m	Steep = high resistance	Digital Elevation Model (DEM) derived from SRTM (Shuttle Radar Topography Mission)	Farr et al., 2007; Gorelick et al., 2017
	Elevation	meters a.s.l.	30 m	—	DEM derived from SRTM	Farr et al., 2007; Gorelick et al., 2017
	Forest Types	categorical (reclassified)	Raster	Reclassified based on habitat type	Thai RFD Land Cover	https://www.forest.go.th/land/
	Distance to Streams	meters	Vector	Optional hydrological variable	Euclidean Distance	http://download.geofabrik.de/asia. html (Planet OpenStreetMap)
	Percent Tree Cover	% canopy cover	250 m	Dense cover = higher resistance	MODIS	Dimiceli (2017)
	TRI (Terrain Ruggedness Index)	ruggedness index	30 m	High = lower mobility	Extracted from digital elevation model (DEM)	Beven and Kirkby (1979)
	TWI (Topographic Wetness Index)	index (unitless)	30 m	Indicates drainage/water accumulation	Extracted from digital elevation model (DEM)	Beven and Kirkby (1979)
Anthropogenic	Distance to Major Roads	meters	Vector	Farther = less accessible	Euclidean Distance	http://download.geofabrik.de/asia. html (Planet OpenStreetMap)

Nighttime Lights	brightness index	500 m	Bright = urban/high resistance	VIIRS	Elvidge et al. (2021)
Degree of Urbanization	categorical (0–3)	250 m	Urban = high resistance	GHS-POP or GHSL	Schiavina et al. (2023)
Residential Population	people per km²	100 m	Proxy for human pressure	WorldPop	WorldPop (2020)

Table S3. Haplotype distribution of mitochondrial DNA D-loop sequences in wild Asian elephants from Phu Khieo (PK) and Khao Ang Rue Nai (ARN) Wildlife Sanctuaries.

Haplotype	N (Total)	Sample ID (PK)	Sample ID (ARN)
TH1	14		ARN00, ARN1, ARN3, ARN4, ARN5, ARN11, ARN147, ARN12, ARN19, ARN98, ARN99, ARN104, ARN108, ARN209
TH2	23	PK38, PK39, PK62, PK63, PK64, PK65, PK66	ARN9, ARN10, ARN93, ARN103, ARN148, ARN149, ARN210, ARN8, ARN102, ARN106, ARN109, ARN110, ARN144, ARN145, ARN146
TH3	1		ARN22
TH4	1		ARN112
TH5	1		ARN15
TH6	1		ARN21
TH7	1	PK11	
TH8	16	PK12, PK20, PK21, PK24, PK33, PK34, PK41, PK44, PK50, PK53, PK55, PK56, PK58, PK59, PK71, PK95	
TH9	1	PK17	
TH10	2	PK22, PK27	
TH11	1	PK23	
TH12	2	PK25, PK26	
TH13	1	PK68	
TH14	1	PK73	

Table S4. AMOVA results for mitochondrial DNA D-loop sequences from wild Asian elephants in Thailand.

Source of variation	df [1]	Sum of squares	Variance components	Percentage of variation	*p*-value
Among populations	1	20.890	0.51516	11.65	0.007
Within populations	64	249.936	3.90525	88.35	
Total	65	270.826	4.42041	100	

df[1] =Degree of freedom.

Table S5. Mismatch distribution parameters and demographic expansion test results for mitochondrial DNA sequences in Phu Khieo (PK) and Khao Ang Rue Nai (ARN) populations.

Parameter	PK	ARN
Mean pairwise differences	7.012	8.032
Tau (τ)	15.033	14.496
Theta0 (pre-expansion)	0.000	0.002
Theta1 (post-expansion)	4.199	1.820
Sum of Squared Deviations (SSD)	0.0845	0.229*
Harpending's Raggedness Index	0.175	0.425*

* Indicates statistical significance at $p < 0.05$.

Table S6. Mantel test results showing correlations (*r*) and *p*-values between genetic distance and environmental resistance variables for Phu Khieo (PK) and Khao Ang Rue Nai (ARN). Significant results ($p < 0.05$) are marked with asterisk (*).

Environmental Variable	Mantel *r* (PK)	*p*-value (PK)	Mantel *r* (ARN)	*p*-value (ARN)
Degree of Urbanization	0.0695	0.1263	0.359*	0.0008*
Distance to Major Roads	0.166*	0.0002*	0.267*	0.0163*
Nighttime Lights	-0.1088	0.0165	0.208*	0.0382*
TRI	-0.0222	0.625	0.181	0.0654
Percent Tree Cover	0.0551	0.226	0.155	0.1082
Distance to Streams	0.090*	0.0477*	0.104	0.1745
TWI	0.1375*	0.0024*	0.072	0.2411
Slope	-0.028	0.5384	0.047	0.2811
Canopy Height	0.0443	0.3306	0.011	0.4517
Elevation	0.0607	0.1817	0.008	0.4802
Forest Types	0.0701	0.1232	0.019	0.5680
Residential Population	-0.0844	0.0633	-0.026	0.6096

Table S7. Results of spatial autocorrelation analyses (LSI) for wild Asian elephants in Phu Khieo (PK) and Khao Ang Rue Nai (ARN) Wildlife Sanctuaries.

Population	Distance class (m)	n	Autocorrelation (Ay)	Upper-tail *p*-value	Lower-tail *p*-value
PK	0–3528	49	0.014	0.894	0.106
	3528–7445	48	0.008*	1.000	0.000
	7445–10264	50	0.017	0.726	0.274
	10264–12595	48	0.020	0.392	0.609
	12595–15094	50	0.019	0.532	0.470
	15094–17152	48	0.022	0.271	0.730
	17152–19007	50	0.028*	0.014	0.986
	19007–21454	47	0.033*	0.000	1.000
	21454–27172	51	0.027	0.060	0.940
	27172–49699	55	0.008	0.999	0.001
ARN	0–277	56	0.093	0.366	0.634
	277–2174	55	0.046	0.637	0.363
	2174–5706	57	0.033	0.652	0.348
	5706–6196	55	0.024	0.655	0.345
	6196–17068	57	0.029	0.891	0.109
	17068–19073	54	0.100	0.348	0.652
	19073–20945	55	0.096	0.376	0.624
	20945–23501	57	0.097	0.292	0.708
	23501–34383	58	0.053	0.580	0.420
	34383–46625	57	0.065	0.097	0.903

* Indicates statistical significance at $p < 0.05$.

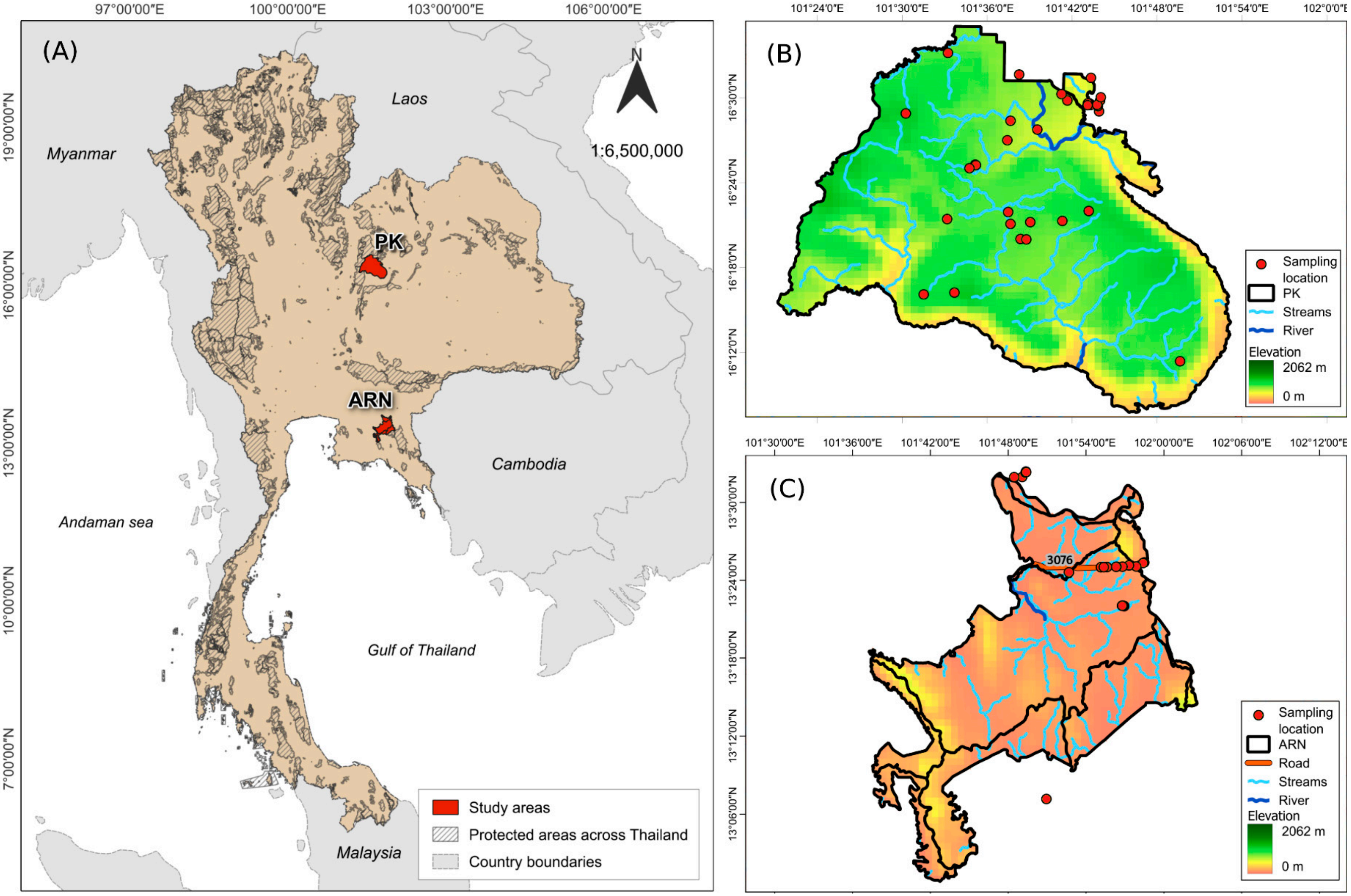

Figure S1. Geographic locations of the two study areas in Thailand. (A) Location of the two study areas in Thailand. (B) Phu Khieo Wildlife Sanctuary (PK) in the northeastern region (C) Khao Ang Rue Nai Wildlife Sanctuary (ARN) in the eastern region.

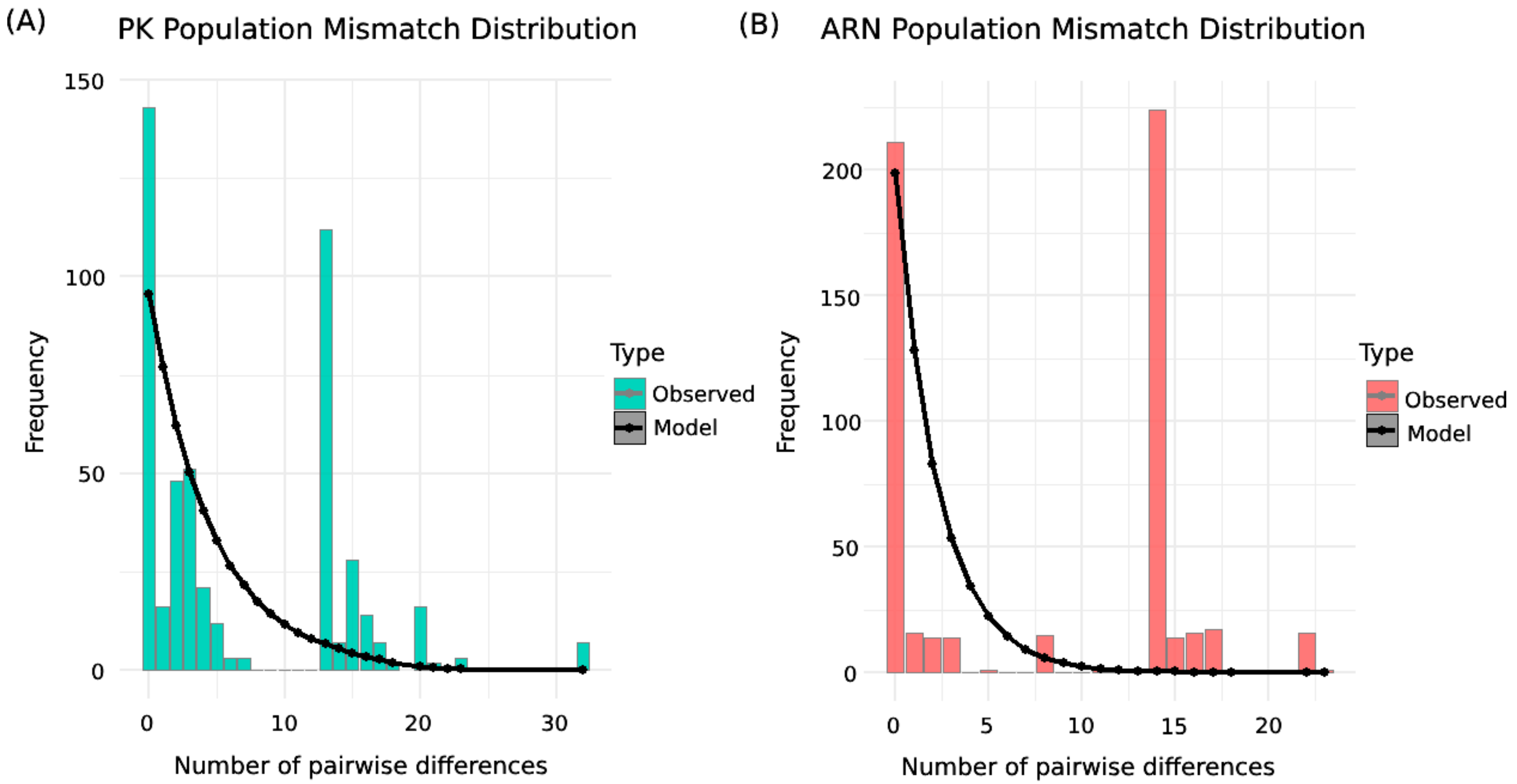

Figure S2. Mismatch distribution of pairwise nucleotide differences under the sudden expansion model. (A) Phu Khieo Wildlife Sanctuary (PK) (B) Khao Ang Rue Nai Wildlife Sanctuary (ARN).

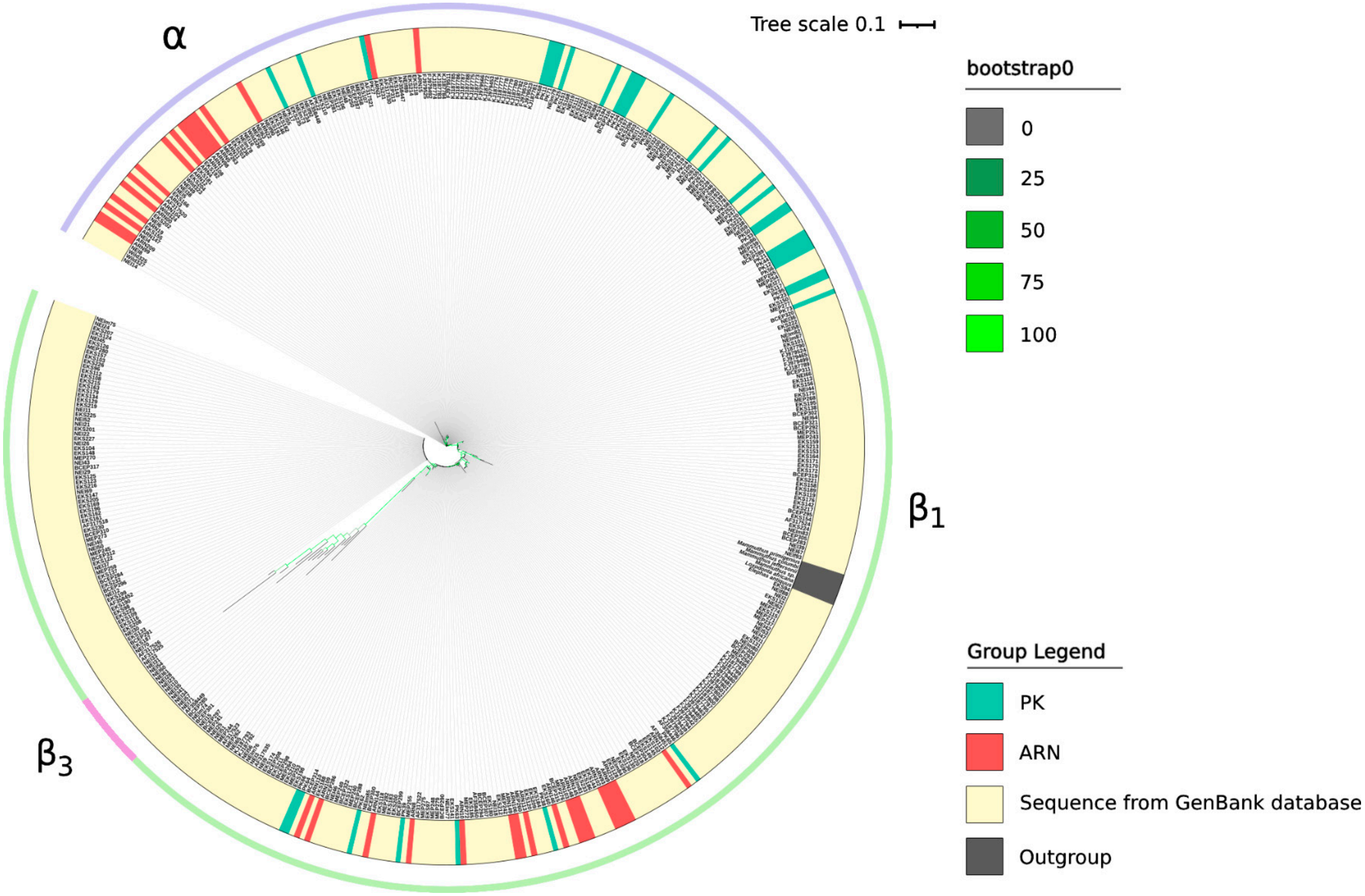

Figure S3. Maximum Likelihood (ML) of mitochondrial DNA D-loop haplotypes from elephants in Phu Khieo (PK) and Khao Ang Rue Nai (ARN). Branch lengths represent relative divergence times.

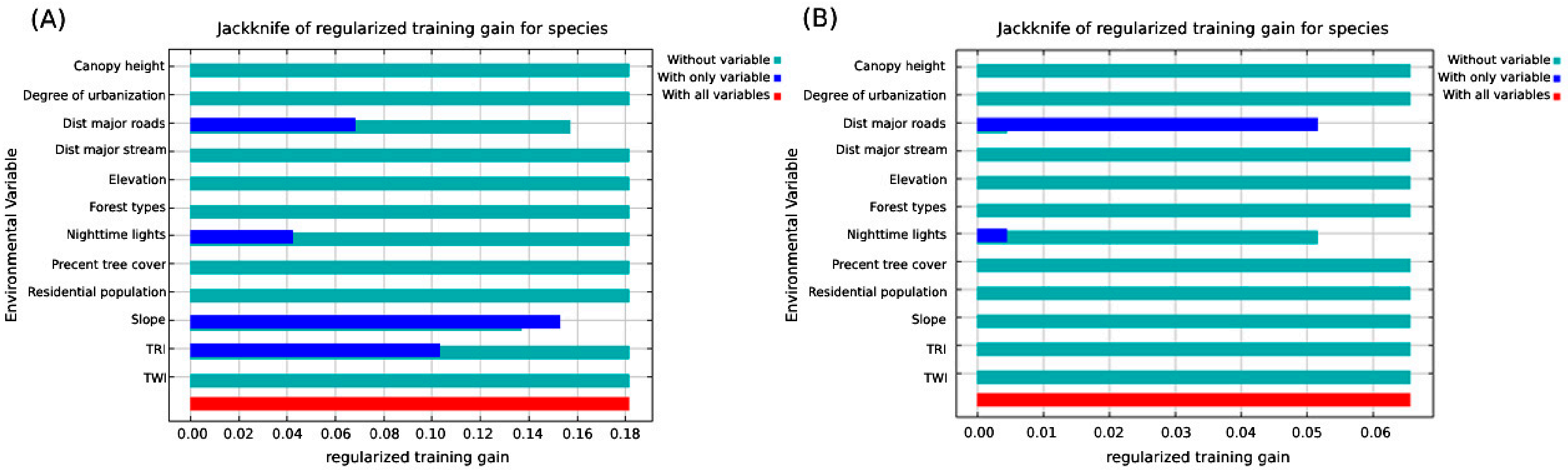

Figure S4. Jackknife of regularized training gain for environmental variables in species modeling**.** (A) Phu Khieo Wildlife Sanctuary (PK) (B) Khao Ang Rue Nai Wildlife Sanctuary (ARN).

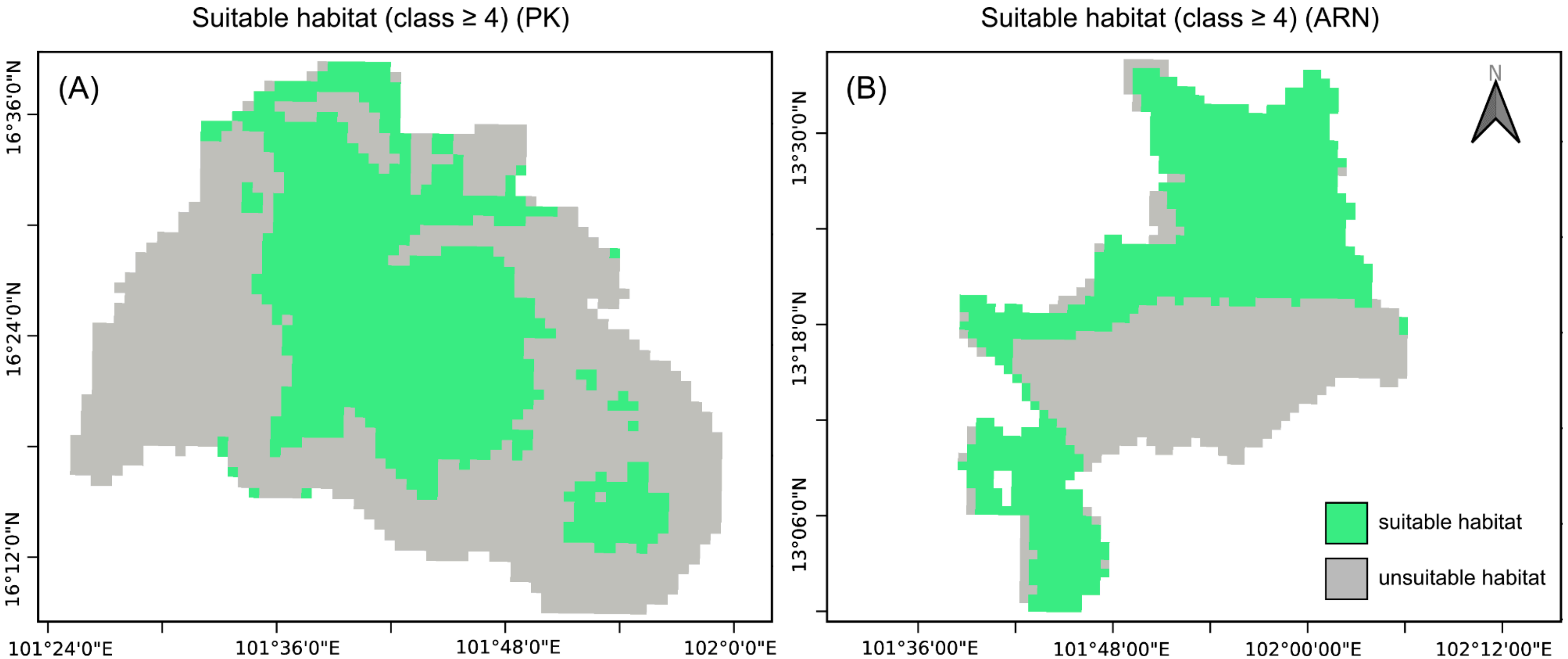

Figure S5. Habitat suitability classification for Asian elephant**.** (A) Phu Khieo Wildlife Sanctuary (PK) (B) Khao Ang Rue Nai Wildlife Sanctuary (ARN). Green indicates suitable habitat (MaxEnt class ≥ 4); gray indicates unsuitable habitat. Models were based on elephant occurrence and environmental layers.

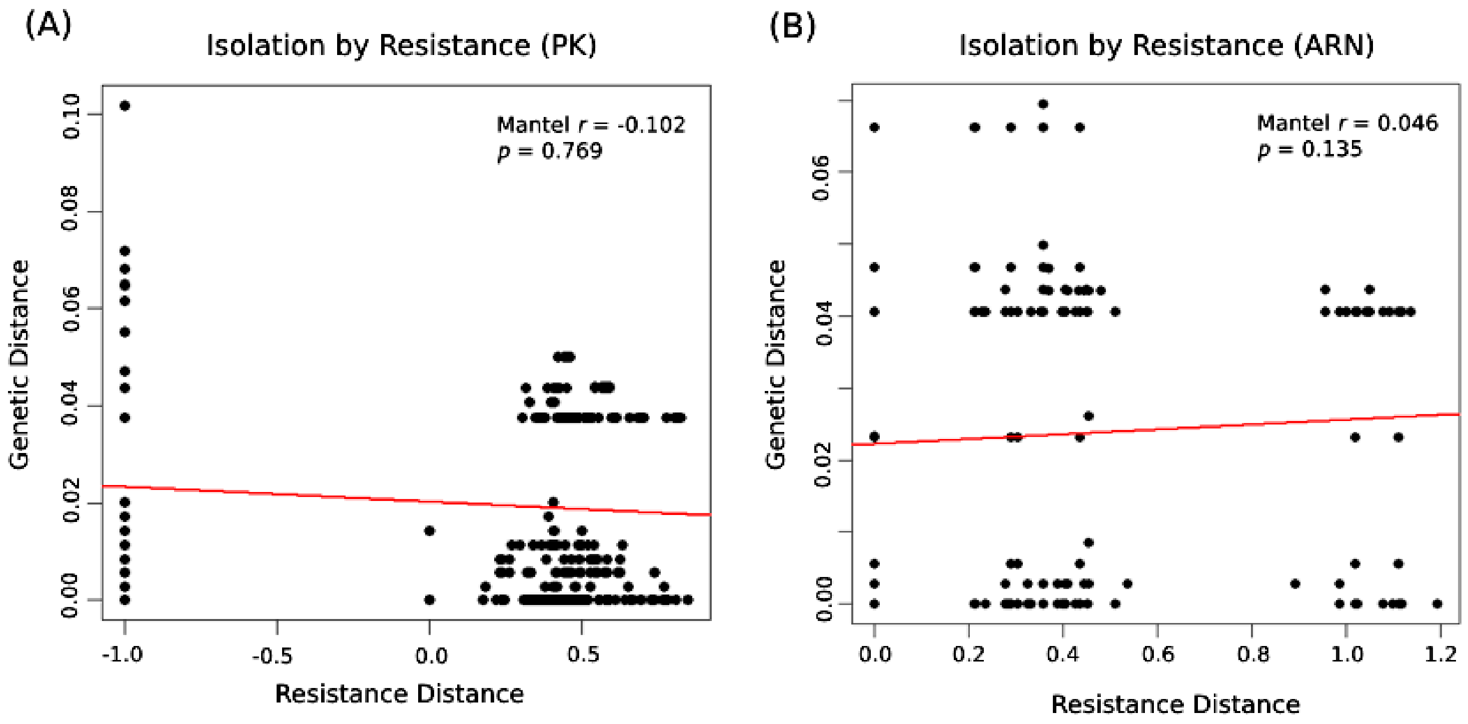

Figure S6. Relationship between resistance distance and genetic distance (Isolation by Resistance) in Asian elephants. (A) Phu Khieo Wildlife Sanctuary (PK) (B) Khao Ang Rue Nai Wildlife Sanctuary (ARN).

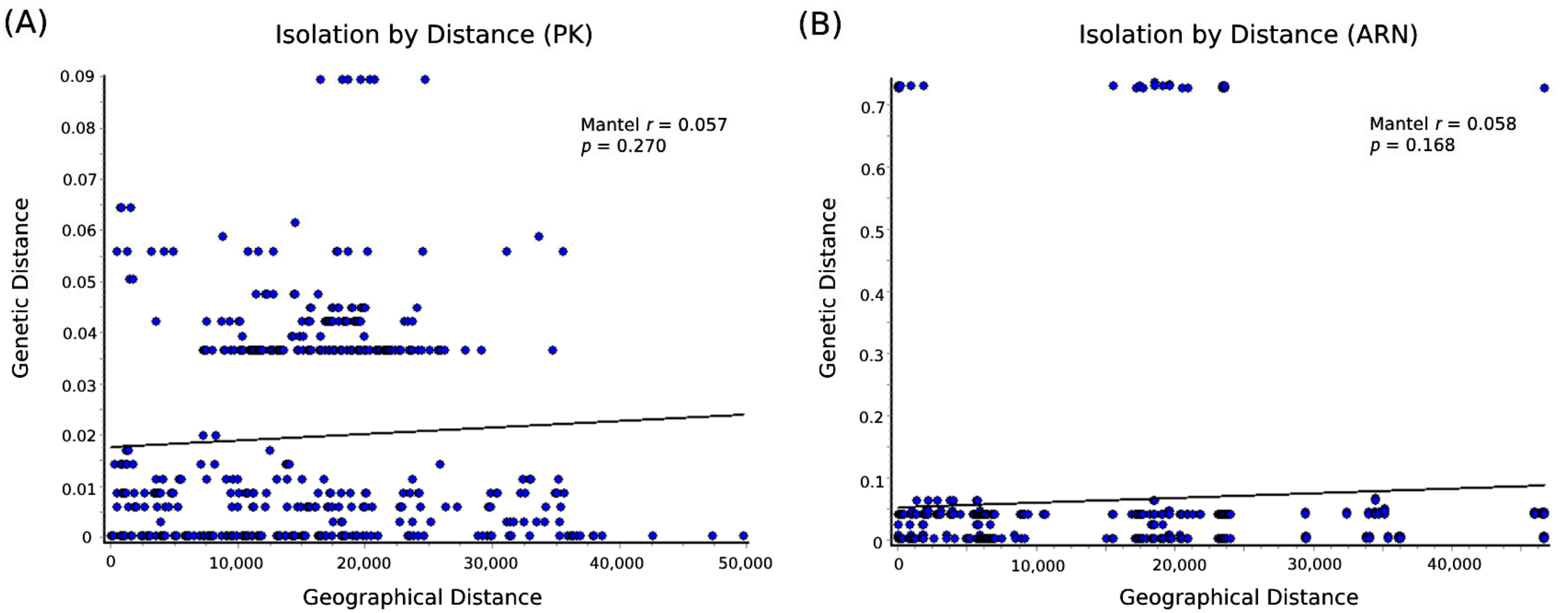

Figure S7. Relationship between geographic distance and genetic distance (Isolation by Distance) in Asian elephants. (A) Phu Khieo Wildlife Sanctuary (PK) (B) Khao Ang Rue Nai Wildlife Sanctuary (ARN).

References

1. Quainoo, D. K., Chalermwong, P., Muangsuk, P., Nguyen, T. H. D., Panthum, T., Singchat, W., Budi, T., Duengkae, P., Suksavate, W., Chaiyes, A., Sanannu,
2. S., Tipkantha, W., Bangkaew, N., Sripiboon, S., Muangmai, N., Han, K., Maneeorn, P., Kaewparuehaschai, M., Leamsaard, G., & Kanchanasaka, C.
3. (2025). Genetic insights for enhancing conservation strategies in captive and wild Asian elephants through improved non-invasive DNA-based individual identification. *PLOS One, 20*(5), e0320480. https://doi.org/10.1371/journal.pone.0320480
4. Ariyaraphong, N., Ho My Nguyen, D., Singchat, W., Suksavate, W., Panthum, T., Langkaphin, W., Chansitthiwet, S., Angkawanish, T., Promking, A.,
5. Kaewtip, K., Jaisamut, K., Ahmad, S. F., Trirongjitmoah, S., Muangmai, N., Taesumrith, O., Inwiset, S., Duengkae, P., & Srikulnath, K. (2022). Standard Identification Certificate for Legal Legislation of a Unique Gene Pool of Thai Domestic Elephants Originating from a Male Elephant Contribution to Breeding. *Sustainability*, *14*(22), 15355. https://doi.org/10.3390/su142215355
6. Fickel, J., Lieckfeldt, D., Ratanakorn, P., & Pitra, C. (2007). Distribution of haplotypes and microsatellite alleles among Asian elephants (*Elephas maximus*)
7. in Thailand. *European Journal of Wildlife Research*, *53*(4), 298–303. https://doi.org/10.1007/s10344-007-0099-x
8. Dejchaisri, S., van Lith, H., Rutten, G. P. A. R., Kumsuk, M., Savini, C., Pattanakaew, P., Chareundong, T., Manopawitr, P., Kiewwan, N., Simon, H.,
9. Colenbrander, B., Stout, A. E. T., Lenstra, A. J., Wajjwalku, W., Bhumpakphan, N., Thongtipsiridech, S., Thitaram, C., Pinyopummin, A., Nitaya, K., ... Kaewket, C. (2008). Cytochrome b haplotypes of wild Asian elephant (*Elephas maximus*) populations in Kaeng Krachan National Park and Phukhieo Wildlife Sanctuary. Paper presented at the 29th Thailand Wildlife Seminar, Bangkok, Thailand, December 18–19, 2008.
10. Lei, R., Brenneman, R. A., Schmitt, D. L., & Louis, E. E. (2011). Genetic diversity in North American captive Asian elephants. *Journal of Zoology, 286*(1),
11. 38–47. https://doi.org/10.1111/j.1469-7998.2011.00851.x
12. Meyer, M., Palkopoulou, E., Baleka, S., Stiller, M., Penkman, K. E. H., Alt, K. W., Ishida, Y., Mania, D., Mallick, S., Meijer, T., Meller, H., Nagel, S.,
13. Nickel, B., Ostritz, S., Rohland, N., Schauer, K., Schüler, T., Roca, A. L., Reich, D., & Shapiro, B. (2017). Palaeogenomes of Eurasian straight-tusked elephants challenge the current view of elephant evolution. *ELife*, *6*. https://doi.org/10.7554/elife.25413
14. Enk, J., Devault, A., Widga, C., Saunders, J., Szpak, P., Southon, J., Rouillard, J.-M., Shapiro, B., Golding, G. B., Zazula, G., Froese, D., Fisher, D. C.,
15. MacPhee, R. D. E., & Poinar, H. (2016). Mammuthus Population Dynamics in Late Pleistocene North America: Divergence, Phylogeography, and Introgression. *Frontiers in Ecology and Evolution, 4*. https://doi.org/10.3389/fevo.2016.00042
16. Kornienko, I. V., Faleeva, T. G., Oreshkova, N. V., Grigoriev, S. E., Grigoreva, L. V., Simonov, E. P., Kolesnikova, A. I., Putintseva, Y. A., & Krutovsky, K.
17. V. (2018). Complete mitochondrial genome of a woolly mammoth (*Mammuthus primigenius*) from Maly Lyakhovsky Island (New Siberian Islands, Russia) and its phylogenetic assessment. *Mitochondrial DNA Part B, 3*(2), 596–598. https://doi.org/10.1080/23802359.2018.1473721
18. Debruyne, R., Chu, G., King, C. E., Bos, K., Kuch, M., Schwarz, C., Szpak, P., Gröcke, D. R., Matheus, P., Zazula, G., Guthrie, D., Froese, D., Buigues, B.,
19. de Marliave, C., Flemming, C., Poinar, D., Fisher, D., Southon, J., Tikhonov, A. N., & MacPhee, R. D. E. (2008). Out of America: Ancient DNA Evidence for a New World Origin of Late Quaternary Woolly Mammoths. *Current Biology*, *18*(17), 1320–1326. https://doi.org/10.1016/j.cub.2008.07.061
20. Lang, N., Jetz, W., Schindler, K., & Wegner, J. D. (2023). A high-resolution canopy height model of the Earth. *Nature Ecology & Evolution*, *7*(11), 1778-1789.
21. Elvidge, C. D., Zhizhin, M., Ghosh, T., Hsu, F. C., & Taneja, J. (2021). Annual time series of global VIIRS nighttime lights derived from monthly averages:
22. 2012 to 2019. *Remote Sensing, 13*(5), 922.
23. Farr, T. G., Rosen, P. A., Caro, E., Crippen, R., Duren, R., Hensley, S., ... & Alsdorf, D. (2007). The shuttle radar topography mission. *Reviews of*
24. geophysics, 45(2).

25. Gorelick, N., Hancher, M., Dixon, M., Ilyushchenko, S., Thau, D., & Moore, R. (2017). Google Earth Engine: Planetary-scale geospatial analysis for
26. everyone. Remote sensing of Environment, 202, 18-27.
27. Schiavina, M., Melchiorri, M., & Pesaresi, M. (2023). GHS-SMOD R2023A-GHS settlement layers, application of the Degree of Urbanisation methodology
28. (stage I) to GHS-POP R2023A and GHS-BUILT-S R2023A, multitemporal (1975-2030). *European Commission, Joint Research Centre (JRC)*.
29. WorldPop. PopulationCounts2000–2020UN-Adjusted Unconstrained100m. 2020. [Data Set]. https://www.worldpop.org/
